# Genomic analysis reveals the role of integrative and conjugative elements in plant pathogenic bacteria

**DOI:** 10.1186/s13100-022-00275-1

**Published:** 2022-08-12

**Authors:** Jéssica Catarine Silva de Assis, Osiel Silva Gonçalves, Alexia Suellen Fernandes, Marisa Vieira de Queiroz, Denise Mara Soares Bazzolli, Mateus Ferreira Santana

**Affiliations:** 1 Grupo de Genômica Evolutiva Microbiana, Laboratório de Genética Molecular de Microrganismos, Departamento de Microbiologia, Instituto de Biotecnologia Aplicada À Agropecuária, Universidade Federal de Viçosa, Viçosa, Minas Gerais Brazil; 2 Laboratório de Genética Molecular de Microrganismos, Departamento de Microbiologia, Instituto de Biotecnologia Aplicada À Agropecuária, Universidade Federal de Viçosa, Viçosa, Minas Gerais Brazil; 3 Laboratório de Genética Molecular de Bactérias, Departamento de Microbiologia, Instituto de Biotecnologia Aplicada À Agropecuária, Universidade Federal de Viçosa, Viçosa, Minas Gerais Brazil

**Keywords:** Genome evolution, Horizontal gene transfer, Mobile genetic elements (MGE), Phytopathology

## Abstract

**Background:**

ICEs are mobile genetic elements found integrated into bacterial chromosomes that can excise and be transferred to a new cell. They play an important role in horizontal gene transmission and carry accessory genes that may provide interesting phenotypes for the bacteria. Here, we seek to research the presence and the role of ICEs in 300 genomes of phytopathogenic bacteria with the greatest scientific and economic impact.

**Results:**

Seventy-eight ICEs (45 distinct elements) were identified and characterized in chromosomes of *Agrobacterium tumefaciens, Dickeya dadantii,* and *D. solani*, *Pectobacterium carotovorum* and *P. atrosepticum*, *Pseudomonas syringae*, *Ralstonia solanacearum* Species Complex, and *Xanthomonas campestris*. Intriguingly, the co-occurrence of four ICEs was observed in some *P. syringae* strains. Moreover, we identified 31 novel elements, carrying 396 accessory genes with potential influence on virulence and fitness, such as genes coding for functions related to T3SS, cell wall degradation and resistance to heavy metals. We also present the analysis of previously reported data on the expression of cargo genes related to the virulence of *P. atrosepticum* ICEs, which evidences the role of these genes in the infection process of tobacco plants.

**Conclusions:**

Altogether, this paper has highlighted the potential of ICEs to affect the pathogenicity and lifestyle of these phytopathogens and direct the spread of significant putative virulence genes in phytopathogenic bacteria.

**Supplementary Information:**

The online version contains supplementary material available at 10.1186/s13100-022-00275-1.

## Background

Integrative and conjugative elements (ICEs) are self-transmissible mobile elements that play a central role in bacterial adaptation processes; hence, they can directly affect the evolution of their host [1, 2]. These widely distributed elements are currently found integrated into the bacterial chromosome, as they are capable of performing their excision, by recombination of direct repeat sequences (DRs) that flank the element (attachment sites), and transference by conjugation, carrying their machinery in a modular structure [3–5]. The main genetic modules found in ICEs include genes that encode functions related to their integration and excision from the host chromosome, conjugation, and regulation. These modules also have variable content, which leads these functions to be performed by different mechanisms from a diverse range of genes [5, 6]. ICEs often carry cargo genes, thus conferring significant phenotypes to the host cell, such as virulence, resistance to antibiotics and heavy metals that are important to bacteria fitness. For instance, ICE_Tn4371_6061 discovered on *Pseudomonas aeruginosa,* provides resistance against carbapenem antibiotics, and ICE*Pm1* of *Proteus mirabilis*, *Providencia stuartii* and *Morganella morgani*, which carry genes that encode an adhesion protease and an iron acquisition system that contributes to the virulence of these bacteria [4, 7–9].

ICEs are broadly distributed in bacterial chromosomes, and some studies demonstrate their presence in plant pathogenic bacteria, which are microorganisms involved in major crop losses by host tissue invasion using virulence factors such as biofilm formation and toxins [3, 10]. For instance, different ICEs were found in strains of *Pseudomonas syringae* pv. *actinidiae* conferring resistance to heavy metals, and ICE HAI2 of *Pectobacterium atrosepticum,* which transmits genes that codify a biosynthetic cluster of an important virulence factor [11, 12]. Among these phytopathogenic bacteria, the most relevant were classified by Mansfield et al., in 2012, according to their scientific relevance and economic impact, in a ranking composed of *Pseudomonas syringae, Ralstonia solanacearum, Agrobacterium tumefaciens, Xanthomonas oryzae pv. oryzae, Xanthomonas campestris, Xanthomonas axonopodis pv. manihotis, Erwinia amylovora, Xylella fastidiosa, Dickeya* (*dadantii* and *solani*) and *Pectobacterium* (*carotovorum* and *atrosepticum*) [13].

A large part of pioneer studies involving ICEs was based only on phenotypes conferred by cargo genes and did not provide broader knowledge about these elements [5]. However, the development of Whole-Genome Sequencing (WGS) efforts leading to the large availability of complete genome sequences has enabled the conducting of investigation to clarify the role of ICEs in bacterial evolution [14–16]. Thus, here we search for ICEs integrated into 300 complete genomes of major phytopathogenic bacteria and analyze putative cargo genes and their potential role in virulence or adaptation.

## Results

### The most comprehensive dataset of ICEs found integrated into the plant pathogenic bacteria genomes

**Fig. 1:**
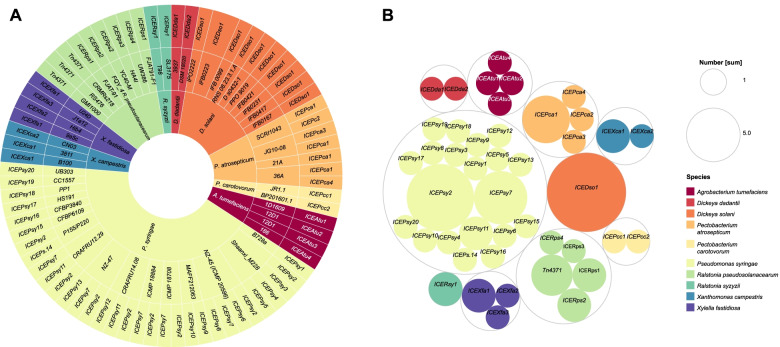
Distribution of ICEs among bacterial strains. Solar explosion chart indicating the elements found in all strains. Bacterial species were arranged from the species with the largest number of elements to the species with the least number of elements and separated by color: Lilac: *P. syringae;* Dark blue: *D. solani*; Light blue: *R. pseudosolanacearum*; Green (from the darkest to the lightest, respectively): *P. atrosepticum, X. fastidiosa, A. tumefaciens, X. campestris*; Yellow: *R. syzigii*; Light orange: *P. carotovorum*; Dark orange: *D. dadantii*. From the inside out of the chart: The bacterial species, name of the strains, and the identified elements present in each strain. **b** Hierarchical organization of ICEs distribution around bacterial species, with color-coding in species as shown in the legend. The scale beside the plot shows the number of ICEs found for each species

**Fig. 2:**
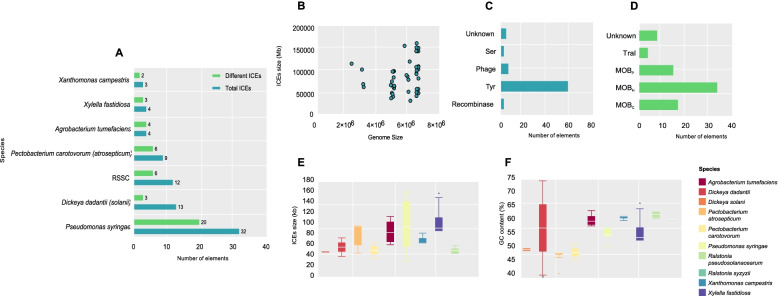
General ICES identification results. **a** Bar chart of ICEs number distribution by groups of bacteria (dark blue: total elements, light blue: different elements); **b** Distribution chart of genome size, in bases pair, compared to the size of ICEs; **c** The type of Integrases found in the ICEs. **d** The type of Relaxase family found in the ICEs. **e** Bar chart of ICEs size by species f) Bar chart of GC content of the ICEs by species

We first sought to identify and analyze ICEs in the genomes of important plant pathogenic bacteria in molecular plant pathology. A total of 78 putative elements were found in nine species of phytopathogenic bacteria, including the species of *D. dadantii, D. solani, P. atrosepticum, P. carotovorum, A. tumefaciens, P. syringae, X. campestris, X. fastidiosa* and *R. solanacearum* species complex (RSSC) (Fig. 1 and Table S11). Among these elements, 45 ICEs were found to be distinct, and a greater number of ICEs were found in the genome of *P. syringae* (33, 20 of which were distinct elements) (Fig. 2A). There were no ICEs identified in the chromosomal sequences of *X. oryzae*, *X. axonopodis*, or *E. amylovora*.

The elements exhibited great variation in sequence size. The largest element *ICEPsy10* was found in *P. syringae* with 161 kb and the smallest *ICEDda*2 was found in *D. dadantii* with 40 kb. Overall, the average size of the elements was 80 kb. The GC content ranged from 40 to 66%, which was found in *ICEPat*2 and *ICEXfa*2, respectively. The average content was 55% (Fig. 2F and Table S12). As already expected, no relationship was found between the increased size of the host genome and the presence of the elements (Fig. 2B).

Most of the elements (88%, n = 68) were found inserted in tRNA genes, whilst nine were found in other genes. Attachment sites (*att*) were identified in 49 ICEs; however, our search methods did not find the *att* sites in any elements integrated into RSSC genomes (Table S13). 58 ICEs encode the Tyr recombinase family, and 34 ICEs encode the MOB_H_ relaxase gene (Fig. 2C, D; Table S14).

Interestingly, we found ICEs with core modular genes seen in other species. The *ICEPca2* from *P. carotovorum* was found in the genome of *Serratia plymuthica* C-1 strain, and comparative analysis demonstrated that the elements share 99% nucleotide similarity (Data not shown). Also, *ICEPca*1 shared 82% of nucleotide identity with a novel ICE from *P. aroidearum* strain L6; *ICEXca*1 shared 88% sequence identity with a novel ICE from *Xanthomonas arboricola* pv. *juglandis* strain Xaj 417. Two ICEs from our dataset, *ICEDda*1 from *D. dadantii* and *Tn4371* of *R. pseudosolanacearum* had been previously classified in the family *Tn4371 *[15, 17]. However, comparative analyses between these two elements indicated low similarity (45% of nucleotide identity).

### ICEs carry genes with potential impact on the pathogenicity and lifestyle of plant pathogenic bacteria

**Fig. 3:**
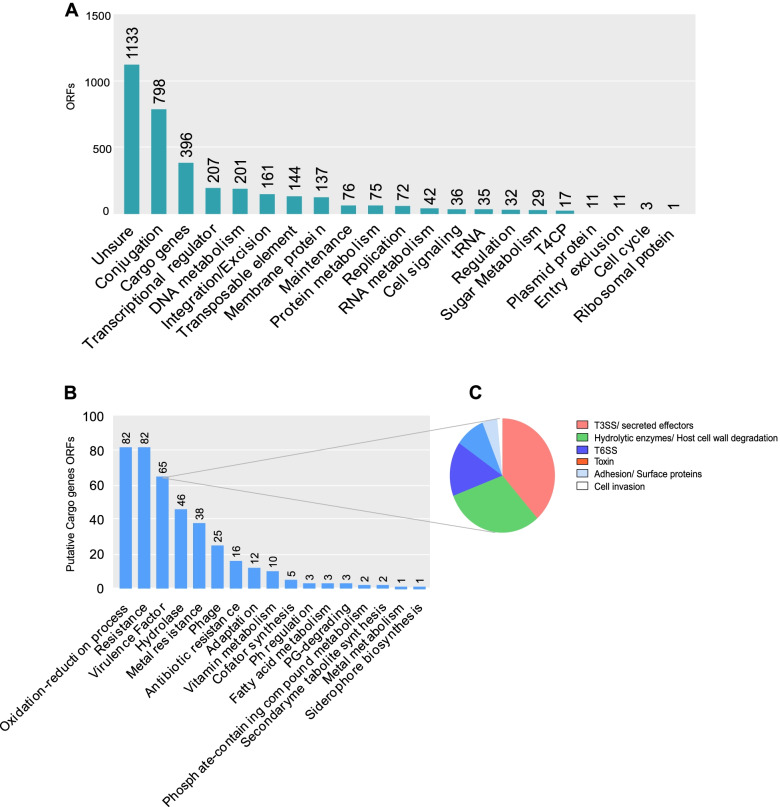
Putative functions of ICEs genes. **a** Bar chart of putative roles codified by ICEs genes separated by categories (Unsure category comprises Hypothetical protein, Domain of unknown function (DUF genes), and genes with undetermined function). **b** Bar chart of Cargo genes divided by putative roles. **c** Pie chart representing putative roles of Virulence factors carried by ICEs

The genes carried by the elements were also investigated and classified for their putative role. As expected, a greater number of hypothetic proteins and genes with unknown functions were identified, followed by conjugation and cargo genes, in general (Fig. 3A). Specifically, among the cargo genes, those encoding functions of oxidation–reduction processes and resistance appeared in a greater number of ORFs (open reading frames) in the elements, followed by virulence factors (Fig. 3B). Putative virulence and adaption roles were verified in most ICEs by the annotation in specific protein databases. Genes codifying virulence functions were tracked down in 28 elements, while with putative adaption, roles were spotted in 12 ICEs, among other important roles, such as metal and antibiotic resistance (Table S15). Regarding virulence, genes were also organized according to their putative function, and the most frequent class of virulence genes were genes that encode proteins translocated by Type III Secretion System (T3SS) and components of this system, followed by genes that encode hydrolytic enzymes involved in host cell wall degradation (Fig. 3C). Altogether, these two sections reveal the existence of ICEs in genomes of high-impact phytopathogenic bacteria and their likely impact on their pathogenicity and lifestyle. Now further, we will present specific results for individual bacteria species.

### ICEs in *Agrobacterium tumefaciens*

Initially, we investigated chromosome sequences of seventeen strains available in the NCBI database, each one of them with two chromosomes. Then, we searched for ICEs in 32 sequences of *A. tumefaciens* chromosomes (Table S3). Our methods allowed the identification of four novel elements in sequences of three strains: *ICEAtu*1 was identified in the chromosome of the strain 1D1609, *ICEAtu*2, and *ICEAtu*3, in the circular and linear chromosomes of the strain 12D1, respectively, and at last, *ICEAtu*4 was identified in the circular chromosome of the strain 186 (Fig. 1). These elements had a mean size of 86 kb; *ICEAtu*3 was the element with the largest sequence (114 kb), followed by *ICEAtu4* (101 kb) and *ICEAtu2* (68 kb), while *ICEAtu1* was the smallest element, with 61 kb of length (Table S12). The mean GC content of the elements was 60% and ranged from 58% (*ICEAtu3*) to 63% (*ICEAtu1*). Only the elements *ICEAtu2* and *ICEAtu3* from the strain 12D1 presented GC content lower than the genome: 58% and 58%, respectively (Table S12). The *att* sites of *ICEAtu3* and *ICEAtu4* were identified, and regarding the integration site, both elements and *ICEAtu2* are inserted in tRNA sequences, and *ICEAtu1* is inserted in the *guaA* gene (Tables S13 and 14). The sequence alignment of the ICEs from *A. tumefaciens* revealed that *ICEAtu2* and *ICEAtu4* share 53% of nucleotide identity, mainly due to the gene clusters that represent conjugation and integration modules based on syntenic analysis (Fig. S1, S2, S3, S4, S5, S6, S7, S8, S9 and S10A).

*A. tumefaciens* ICEs encode proteins that may have important functions for pathogenicity, such as cysteine hydrolase and glycosidase in *ICEAtu1*, and Endo-1,4-beta-xylanase in *ICEAtu4*. Likewise, Alkene reductase and glutathione S-transferase on *ICEAtu1* and universal stress protein on *ICEAtu3* may have a putative role in the adaption of these bacteria. We also identified genes coding for a L.D – transpeptidase of *ICEAtu3* and MBL fold metalo-hydrolase in *ICEAtu4,* which provides putative resistance to antibiotics (Table S15).

### ICEs in *Dickeya (dadantii and solani)*

We search for ICEs in chromosomal sequences of thirteen *Dickeya* genomes, two of which are from *D. dadantii*, and eleven genomes belong to the *D. solani* species (Table S9). In those sequences, we were able to find three distinct elements, and a total of thirteen ICEs: *ICEDda1* was found in the chromosomal sequence of the strain 3937 and *ICEDda2,* in the chromosome of the strain DSM 18,020. Interestingly, *ICEDso1* was present in all strains of *D*. *solani*, hence, all investigated strains harbor ICEs (Fig. 1). Among these elements, only *ICEDda1* has been cited in the literature, as an element of the *Tn4371* family (Table S16) [17].

**Fig. 4:**
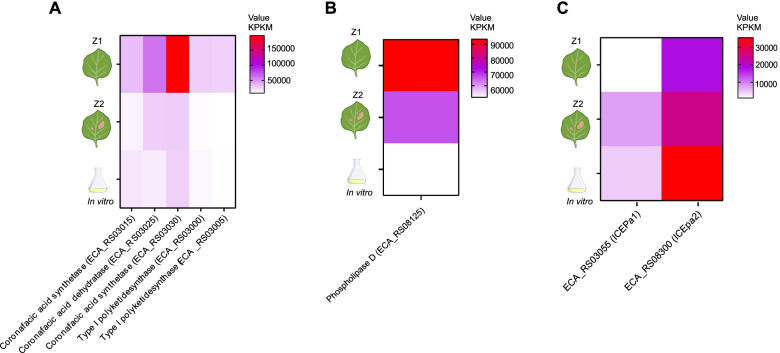
Different Expression Analyses—RPKM Heatmaps: Significant genes of *P. atrosapticum* elements *ICEPa1* and *ICEPa2* (SCRI1043 isolate) in tobacco plants. **a** Coronafacic acid biosynthesis gene cluster carried by *ICEPca1*. **b** Cargo gene of *ICEPca2* with putative Virulence role – Phospholipase D. **c** Relaxases of *ICEPca1* and *ICEPca2*. Scaled expression values are color-coded, and the red color represents high expression. Abbreviation: Z1: Asymptomatic zone, Z2: Symptomatic zone

Regarding the size of those elements, *ICEDda1* was the biggest element, with 74 kb, followed by *ICEDso1* (48 kb), while *ICEDda2* was the smallest, with 40 kb (Table S12). The GC content of all elements was lower than the content of the genomes, with a mean of 51%, so 52% were in *ICEDso1*, 52% in *ICEDda2,* and 49% in *ICEDso1* (Table S12). Both *ICEDda1* and *ICEDso1* had their attachment sites identified and are inserted in tRNA sequences, and *ICEDda2* was inserted in *bamE* gene (Tables S13 and 14). The comparison among these elements revealed low similarity between each other (< 50% of nucleotide identity) (Fig. 4B). However, clusters of syntenic genes were observed between all three elements, which were related to recombinase genes, some transcriptional regulators and cargo genes related to the Type VI secretion system (T6SS). Furthermore, *ICEDda1* and *ICEDso1* may also share their conjugation and regulation modules, since syntenic gene clusters comprise genes of type IV secretion system (T4SS) and a toxin-antitoxin system were verified (Fig. S2). Among the main cargo genes of these ICEs, we identified a putative tellurium resistance (*terB*) and an entry exclusion (*eexN*) in *ICEDda1*. Moreover, component genes of T6SS, *hcp* effectors, and *tssI—vgrG* with putative virulence roles were verified in all three different elements (Table S15).

### ICEs in *Pectobacteriumcarotovorum* (and *atrosepticum*)

Eight genomes from *Pectobacterium* were investigated, four from *P. carotovorum* (Pcc) and four from *P. atrosepticum* (Pca). In total, nine elements were identified, six of which were distinct ICEs (Fig. 1, Table S10). In Pcc, we found *ICEPcc1* and *ICEPcc2* in genomes of the strains JR1.1 and BP201601.1, respectively. We observed several co-occurrences in the genome of *Pectobacterium*, including two ICEs (*ICEPca1* and *ICEPca2*) in the chromosome of SCRI1043, the ICEs (*ICEPca1* and *ICEPca3*) in the strain JG10-08, and the elements *ICEPca1* and *ICEPca4* in the genome of the strain 36A. Here, we identified five novel elements for the genus, and only *ICEPca1* had been previously reported [12, 18, 19] (Fig. 1, Table S16).

The *Pectobacterium* ICEs presented similarity between *ICEPca2* and *ICEPcc1* with 69,5% of nucleotide identity, while between *ICEPca2* and *ICEPca4,* it was 68,9% (Fig. S10C). Syntenic analyses revealed that most elements appear to have similar conjugation modules (high identity), except for *ICEPca1*, a highly syntenic gene cluster comprising conjugation and T4SS genes, and similarity between integration genes for *ICEPcc1*, *ICEPca2*, *ICEPcc2* and *ICEPca4* (Fig. S3). Regarding the main cargo genes of these elements, the coronafacic acid biosynthesis cluster of *ICEPca1* is well-known for the virulence in SCRI1043 isolate [12]. Genes encoding proteins with putative virulence roles were also found in *ICEPca2* (Phospholipase D), *ICEPca4* (Arginase family protein) and *ICEPcc2* (Glutathione peroxidase). Moreover, we identified putative entry exclusion genes in *ICEPca2, ICEPca4,* and *ICEPcc1*. Lastly, genes encoding proteins that may confer antibiotic resistance were observed: MBL fold metallo-hydrolase in *ICEPca3* and *ICEPcc2*, and *mipA*/*ompV* family protein in *ICEPca4* (Table S15).

Our analysis of differential gene expression showed a higher expression of the gene cluster of coronafacic acid biosynthesis carried by *ICEPca1* during the asymptomatic stage of *P. atrosepticum* SCRI1043 infection in tobacco, represented by a higher RPKM value in comparison with the symptomatic stage and in vitro culture (Fig. 4), as presented by [20]. Also, the expression of its putative virulence gene, phospholipase D, was verified, with the same pattern displayed by the genes of *ICEPca1* and higher RPKM value in the asymptomatic stage of infection (Fig. 4). Finally, we analyzed the expression of relaxase genes carried by *ICEPca1* (loci tag ECA_RS03055) and *ICEPca2* (loci tag ECA_RS08300) to indicate the stage of infection where the mobilization of these elements may occur. A different pattern of results was verified, and the relaxase of *ICEPca1* presented greater expression in the symptomatic stage of infection, followed by the in vitro culture, and lower expression in the asymptomatic stage (Fig. 4). The relaxase of *ICEPca2* exhibited an upregulation of the expression in the in vitro culture, followed by the expression in the symptomatic phase (Fig. 4).

### ICEs in *Pseudomonas syringae* pathovars

The greatest number of ICEs was found in the *Pseudomonas pathovars*, possibly due to several genomes available for the species. Twenty-eight chromosomes were investigated for ICEs, and 33 elements were found, including 20 distinct elements (Fig. 1, Table S1). Regarding the distribution of these elements in bacterial genomes, *ICEPsy2* was the most common element, present in nine strains, followed by *ICEPsy7,* which was detected in seven strains (Fig. 1). We were able to track down seven *P. syringae* ICEs already cited in the literature. Therefore, this work brings thirteen novel putative elements (Table S16).

**Fig. 5:**
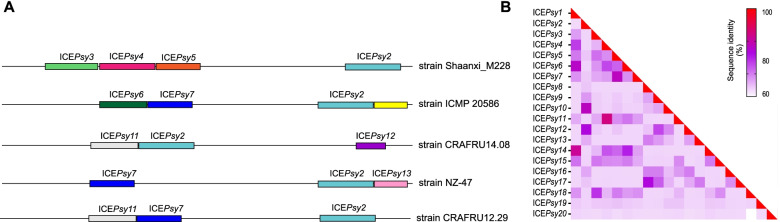
*P. syringae* ICEs. **a** *P. syringae* ICEs co-occurrence and *in-tandem* configuration**.** (White rectangles: bacterial chromosomes – small colored rectangles: ICEs, the colors represent different elements); **b** *P. syringae* ICEs identity matrix heatmap: red—high identity, purple -intermediate white—low identity

Interesting events of ICE co-occurrence have been verified in *P. syringae* chromosomal sequences, mainly in the strains Shaanxi_M228 and ICMP 20,586, each with four monopartite elements uncovered. Furthermore, we spotted the co-occurrence of three monopartite ICEs in genomes of CRAFRU14.08, NZ-47 and CRAFRU12.29; and two elements in MAFF212063, ICMP 18,708, ICMP 18,884 and P155 (Fig. 1). Some of those elements could be observed grouped *in tandem* on the chromosome sequence with the same *att* sites, whose arrangement was outlined in Fig. 5A. This may be the first time in literature that more than three elements are tracked down in one genome.

The size of *P. syringae* elements ranged from 30 kb in *ICEPsy20* to 161 kb in *ICEPsy10*, with a mean of 99 kb (Table S12), and GC content means of 55%, ranging from 50 to 57% (Table S12). Attach sites of fifteen elements were identified, and all the 32 elements were integrated next to a tRNA sequence (Tables S13 and 14). Among the elements, a greater similarity was observed between *ICEPsy4* and *ICEPsy11,* with 85% nucleotide identity followed by *ICEPsy1* and *ICEPsy14,* with 82% of nucleotide identity (Fig. 5B). Comparative analysis of gene clusters illustrated highly syntenic ICEs in *P. syringae* and enabled us to separate these elements into three groups, based on clusters of syntenic genes. Group 1 comprises *ICEPsy3*, *ICEPsy15*, *ICEPsy16*, *ICEPsy18* and *ICEPsy19*. These elements mostly share conjugation and integration modules, as well as a few IS sequences (Fig. S4). Group 2 is the largest and comprises *ICEPsy1*, *ICEPsy4*, *ICEPsy6*, *ICEPsy9*, *ICEPsy11*, *ICEPsy13*, *ICEPsy14*, *ICEPsy17* and *ICEPsy20*; this group shares not only the conjugation and integration modules, as observed in group one, but also cargo genes encoding relevant functions, such as copper and arsenic resistance gene clusters among other cargo genes, except for *ICEPsy20*, the most distinct element among the *P. syringae* ICEs, which shares only an integrase and a maintenance gene (*ardR*) (Fig. S5). Finally, group 3 comprises *ICEPsy2*, *ICEPsy5*, *ICEPsy7*, *ICEPsy10* and *ICEPsy12*; all elements also share the main modules (integration and conjugation). Moreover, *ICEPsy2* and *ICEPsy10* also had a syntenic gene cluster that contains T3SS effectors, a cellulase, a transporter gene cluster and chemotaxis gene clusters that may be involved in element regulation as other cargo genes. This comparison analysis allowed us to verify sequence differences between *ICEPsy2* in the strains ICMP20586 and Shaanxi_M228, due to IS insertions in the elements (Fig. S6).

A large number of cargo genes encoding putative significant roles were identified in *P. syringae* ICEs, mainly copper and arsenic resistance gene clusters of *ICEPsy8* and *ICEPsy13,* which have been described in the literature (Colombi et al., 2017b). However, here we report those gene clusters in *ICEPsy1* and *ICEPsy14* (Cooper and arsenic resistance), *ICEPsy4* and *ICEPsy11* (only Arsenic resistance). A great number of genes encoding proteins with virulence roles were found in some *P. syringae* elements, such as T3SS effector genes, which were found in *ICEPsy2*, *ICEPsy5*, *ICEPsy7*, *ICEPsy9*, *ICEPsy10*, *ICEPsy12* and *ICEPsy18*. Cargo genes conferring putative antibiotic resistance were also identified, such as the *crpP* family protein, which is carried by twelve elements, and *mupB* of *ICEPsy18*. Moreover, a gene cluster encoding putative Tellurium resistance was identified in *ICEPsy2* (Table S15).

### ICEs in *Ralstonia solanacearum* species complex

The chromosome sequences of one hundred *R. solanacearum* strains were examined, revealing seven unique ICEs (six found in *R. pseudosolanacearum* genomes and one found in *R. syzygii*) (Fig. 1, Table S2). Most of those elements had already been described in another work of our research group [15] (Table S16), and here we report the presence of a known element (*ICERps1*) in two more strains, FJAT91-F1 and FJAT91-F8, and a novel element, *ICERps4*, detected in UW386. *ICERps4* has a size of 56.3 kb, 62,4% of GC content, and is inserted in a tRNA sequence (Tables S12, S13 and S14). The alignment of the sequences revealed similarity between *ICERps4* and *ICERps1*, *ICERps2* and *ICERps3,* with nucleotide identity percentages of 79%, 81%, and 78%, respectively (Fig. S3D). It was illustrated with syntenic gene clusters between these elements comprising the conjugation module and other cargo genes (Fig. S7). These elements carry cargo genes that encode a putative role in bacterial adaption, such as Glutathione S-transferase as the element Tn*4371* and *ICERps3*, and gamma-glutamylcyclotransferase carried by *ICERps1*. Likewise, we were able to identify genes with a putative effect on virulence, such as amidohydrolase from *ICERsy1*, and SDR family oxidoreductase, present in Tn*4371* and *ICERps3*. Lastly, the *ICERps1* also carries a Superoxide dismutase gene with a putative antioxidant resistance role (Table S15).

### ICEs in *Xanthomonas campestris* pathovars

We started our research with chromosomal sequences of eighteen *X. campestris* strains, in which we track down two different ICEs, which are two novel elements (*ICEXca1* and *ICEXca2*). *ICEXca1* was found in the chromosomes of B100 and 3811 strains and *ICEXca2* in the chromosome of CN03 (Fig. 1, Table S5). The size of the element *ICEXca1* varied on the different strains: 64.1 kb in B100 and 64.5 kb in 3811. Moreover, the size of the *ICEXca2* element was 83.7 kb; the GC content was 61% and 60%, respectively (Table S12). The attachment sites of the elements were described, and both were inserted in tRNA sequences (Tables S13 and S14). The elements shared low similarity, with a nucleotide identity percentage of 47% (Fig. S10E), however, they have highly syntenic gene clusters (Fig. S8). Both elements identified in *X. campestris* carry cargo genes encoding putative roles in virulence. In ICE*Xca1*, we find genes encoding an aminotransferase and a lytic murein transglycosylase in ICE*Xca2*. This element also carries two putative avirulence effector genes and cargo genes, such as Inositol hexakisphosphate, which may be related to the adaption of these bacteria (Table S15).

### ICEs in *Xylella fastidiosa*

The analysis of chromosomes of twenty-one *X. fastidiosa* strains resulted in the discovery of three distinct ICEs from a total of four elements (Fig. 1, Table S8). The size of the element ranged from 88 kb in *ICEXfa1* to 158 kb in *ICEXfa3,* and the mean GC content was 56 ranging from 54 to 66 (Table S12). The insertion of the element *ICEXfa1* diverged from the others since this element was found integrated next to the ABC transporter gene; *ICEXfa2* and *ICEXfa3* were found integrated next to tRNA sequences. Moreover, only the attachment sites of *ICEXfa2* were identified (Tables S13 and S14). So far, only *ICEXfa2* in the strain Hib4 has been identified in the literature [21]. Hence, our work presents two novel elements in *X. fastidiosa*. The alignment analysis indicates low similarity between the elements with nucleotide identity percentage less or equal to 42% (Fig. S10F). Gene cluster comparison analyses were able to demonstrate significant syntenic groups shared by *ICEXfa3* and *ICEXfa1*, comprising their conjugation and maintenance modules, represented by T4SS genes and a toxin-antitoxin gene cluster, in addition to other cargo genes; *ICEXfa2* exhibits low similarity with the other elements in this analysis as well (Fig. S9). Cargo genes with putative roles in virulence were found in all three *X. fastidiosa* elements, including unidentified virulence factors that can be found in *ICEXfa1* and *ICEXfa3*. Also, genes conferring a putative antibiotic resistance were found in both elements (MBL fold metallo-hydrolase- *ICEXfa1*) and (UDP-3-O-(3-hydroxymyristoyl- glucosamine N-acyltransferase—*ICEXfa3*). We identified genes conferring putative antioxidant resistance (Superoxide dismutase) and a putative role in adaption, such as coproporphyrinogen III oxidase in *ICEXfa2*. Furthermore, a putative Entry exclusion gene was identified in *ICEXfa3* (Table S15).

### The conservation and evolutionary history of plant-pathogen ICEs core-genes

**Fig. 6:**
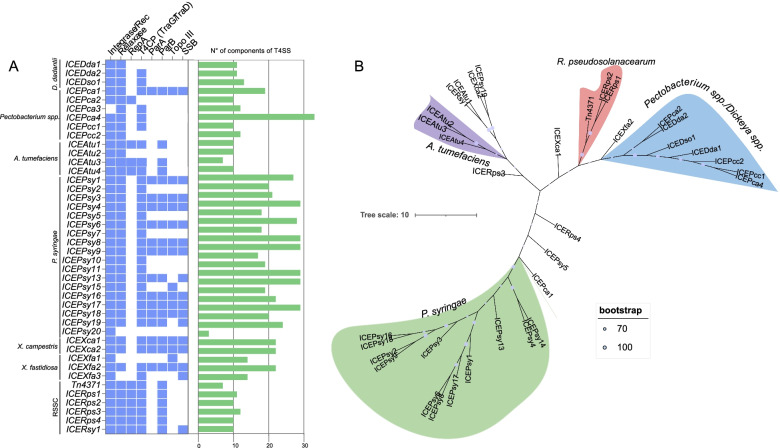
ICEs core-gene conservation and evolutionary history. **a** Plot of eight core genes found in ICEs dataset with the number of components of T4SS grouped by species. Blue colored square indicates the presence of the gene in the element and the colorless square indicates the absence. Abbreviation for Rec, Recombinase; T4CP, type-IV coupling proteins; topo III, topoisomerase III; Single-strand DNA-binding protein (SSB). **b** Maximum Likelihood tree based on the eight backbone gene alignments. The General Time Reversible model and a bootstrap confidence value of 1,000 were applied to the tree. The alignment and phylogenetic analysis were done using MEGA X. The tree is drawn to scale, with branch lengths measured in the number of substitutions per site

Beyond cargo genes carried by the ICEs, we also present the conservation and evolutionary history of ICEs' core genes, which may play a role in element movement. Integrase/recombinase, relaxase, type-IV coupling proteins (T4CPs), ParA, ParB, topoisomerase III, and Single-strand DNA-binding protein (SSB) were among the most conserved core genes in our dataset (Fig. 6A). In addition, we found that ICEs from the same species have common core genes, including their presence/absence and the average of T4SS components, and those lacking the majority of the core genes may be defective elements, such as *ICEPsy20* from *P. syringae* (Fig. 6A). *P. syringae* ICEs had the highest T4SS component average (22 genes set). Next, we group these core genes from putative intact elements and constructed a tree based on the concatenation of eight backbone gene alignments. The species-related ICEs were clustered into four groups on the phylogenetic tree. *P. syringae* ICEs, the most conserved cluster, *A. tumefaciens*, *R. pseudosolanacearum*, and a cluster including ICEs from *Pectobacterium spp*. and *Dickeya spp*. (Fig. 6B). These findings might imply that these elements have the same common origin. Nevertheless, we found clusters with different species, suggesting that the ICEs genes themselves are conserved (Fig. 6B).

## Discussion

The limited understanding regarding ICEs has been overcome in recent years due to the increased availability of whole genome sequences [4, 5, 22]. Some studies have addressed the presence of ICEs in phytopathogenic bacteria but have not dealt in-depth. However, it is necessary to fully understand the relationship between ICEs and economically important plant pathogenic bacteria [3, 10]. Through an *in-silico* investigation of ICEs using 300 genomes available at NCBI, we have identified and characterized 78 putative ICEs integrated into the genomes of the top ten phytopathogenic bacteria. As expected, most ICEs were found integrated into tRNA sequences [4]. These elements encoded core modular genes, including genes that allow Integration/excision modules, conjugation, maintenance and regulation modules [4]. In general, evidence of ICEs features such as GC content indicates a slightly lower value than expected in the host genome, suggesting that these elements may have been acquired recently and probably be under selective pressure to adapt to the codon host’s codon usage [23, 24]. Some elements also carry entry exclusion genes (eexn and traG), which can avoid redundant transfer of ICEs leading to host energy savings [25, 26]

We found no ICEs in *X. oryzae* pv. oryzae*, X. axonopodis* or *E. amylovora,* possibly due to a bias in the software system used, since we are dealing with highly diverse elements and the number of complete genomes available for these species. However, we search for ICEs in 83 complete genomes of *X. oryzae* pv. oryzae and, despite its large repertoire of insertion sequences [27, 28], no ICE was found. Similarly, we investigate 100 sequences of RSSC complete genomes, 40 new genomes more than those analyzed by [15], and one novel ICE (*ICERps4*) was reported for RSSC. We hypothesize that the present variation of ICEs in genomes of different bacterial species may be related to a type of mechanism to control the entry of these elements into cells, which can be more or less rigorous, thus allowing or not the acquisition of ICEs by host cells.

Most of the identified elements were found to carry genes with putative functions for adaptation and virulence. Putative antibiotic resistance genes were also identified, which can be related to the ICEs maintenance module, although they have not been classified in the same way. Regarding the virulence factors found, an increased number of T3SS ORFs was observed in ICEs. This secretion system is an important virulence trait for phytobacteria that allows the translocation of effector proteins into plant cells, either by changing their metabolism or suppressing defenses [29, 30]. Furthermore, we also found genes that encode hydrolytic enzymes involved in host cell wall degradation as the second most prevalent group of virulence factors in our ICEs. Thus, our work highlights the importance of ICEs for a possible function in parasitism evolution. Conserved core genes found in the ICEs may contribute to the element’s mobility and enhance the spread of significant putative virulence genes in phytopathogenic bacteria.

In conclusion, our results suggest a putative association between ICEs and plant pathogen bacteria fitness. Our in silico study opens the doors to further experimental investigations that should be carried out to improve knowledge about the role of ICEs and their cargo genes in phytopathogenic bacteria.

## Materials and methods

### Data

Three hundred complete genomes of phytopathogenic bacteria of economic and scientific impact [13] were downloaded from the National Center for Biotechnology Information (NCBI—http://ftp.ncbi.nih.gov/assembly) in July 2020. The chromosome sequences in GenBank format were used to search for ICEs (Tables S1, S2, S3, S4, S5, S6, S7, S8, S9 and S10).

### Identification and the delimitation of integrative and conjugative elements

To uncover sequences of ICEs in bacterial chromosomes, we resort to a method similar to that applied by Gonçalves and Santana [16]. Thus, we perform a search of the element sequences using BLASTn [31] against known ICEs deposited in the ICEberg database [32, 33], and only sequences that obtained an E-value less or equal to 10^–5^ and coverage more or equal to 50% were selected. We submit the nucleotide sequences of ICEs to the ICEfinder software system (https://db-mml.sjtu.edu.cn/ICEfinder/ICEfinder.html), an online tool provided by ICEberg 2.0 that identifies signature features of integrative and conjugative elements as integrase gene, T4SS, and directed repeats sequences (DRs) in bacterial genomes [32, 33], OriTfinder [34] (https://tool-mml.sjtu.edu.cn/oriTfinder/oriTfinder.html) that identifies transfer origin sequences in bacterial chromosomes, among other features, which indicates the presence of the ICE, MOBscan (https://castillo.dicom.unican.es/mobscan/) that identifies relaxase MOB families, the CONJscan module of MacSyFinder (https://galaxy.pasteur.fr) that identifies conjugative systems in bacterial genomes searching for Type IV secretion systems [35]. We also execute a manual search on the sequences of complete and annotated chromosomes looking for ICEs signature genes: genes that are part of the T4SS involved in the conjugative transfer, such as *tra*, *vir0,* or *trb,* and integrase (*int*) [5]. The attachment regions (*att* site) that provided upper and lower boundaries of the elements were identified in ICEfinder, but when the *att* regions were not detected, we manually identified them using BLASTn. We initially delimited the upper bound of the element by looking for genes of integrases close to tRNAs, then, that region between these genes was selected to make the BLAST look for a repeated sequence in another position of the genome that was close to the coordinates the possible final portion of the element given by ICEfinder. ICEs were named following patterns already described [36].

### The characterization of carrying genes

The annotation of protein sequence was performed using Uniprot (https://www.uniprot.org/) [37], and Pfam (http://pfam.xfam.org/) [38] protein databases. Putative functions of accessory genes were investigated by performing a BLASTp against Pathogen-host Interactions database (http://www.phi-base.org/) [39], Virulence Factor Database (http://www.mgc.ac.cn/VFs/) [32, 33] and Type III secretion system effectors database (http://effectors.bic.nus.edu.sg/blast.php) [40]. The parameters used to identify sequence coding proteins were: e-value less or equal to 10^–5^ and amino acid identity greater than 30% [15]. Subsequently, the nucleotide sequences of the identified elements were downloaded in GenBank format and analyzed using the Geneious® software system (Biomatters Ltd.) for the characterization of the excision and integration, conjugation, regulation and maintenance modules.

### Differential expression analysis

The differential expression analysis was performed using RNAseq data from *P. atrosepticum* isolate SCRI1043 corresponding to two stages of infection in the tobacco plant (asymptomatic and symptomatic) and an in vitro culture. The data are available in NCBI BioProject (accession number PRJNA403794) [20]. The Geneious software system was used for the analysis, following the Expression Analysis tutorial with default parameters. In order to study the gene expression of the ICEs present in the genome, the element sequence was used as a reference to map the reads, and the differential expression was measured. Reads per kilobase per million (RPKM) values were plotted in GraphPad Prism version 8.4.3 to generate heatmaps.

### Comparative analysis

Nucleotide sequences in fasta format of the ICEs identified were submitted to ClustalW [41] to generate Pairwise Identity Matrices for Heatmaps creation, using GraphPad Prism version 8.4.3 for Windows. The sequences were also submitted to Mauve [42] and clinker clustermap.js [43] to generate gene cluster comparison and synteny analyses. Core genes were aligned in ClustalW [44] and concatenated in Mesquite software Version 3.70 (http://www.mesquiteproject.org). The maximum likelihood tree was aligned and constructed using MEGA X [45] based on the eight backbone gene alignments. The General Time Reversible model and a bootstrap confidence value of 1,000 were applied to the tree.

## Supplementary Information

**Additional file 1: Supplementary Table 1.** Genomes of Pseudomonas syringae analyzed in this work. **Supplementary Table 2.** Genomes of Ralstonia solanacearum Species complex analyzed in this work. **Supplementary Table 3.** Genomes of Agrobacterium tumefaciens analyzed in this work. **Supplementary Table 4.** Genomes of Xanhtomonas oryzae pv. oryzae analyzed in this work. **Supplementary Table 5.** Genomes of Xanthomonas campestris analyzed in this work. **Supplementary Table 6.** Genomes of Xanthomonas axonopodis analyzed in this work. **Supplementary Table 7.** Genomes of Erwinia amylovora analyzed in this work. **Supplementary Table 8.** Genomes of Xylella fastidiosa analyzed in this work. **Supplementary Table 9.** Genomes of Dickeya (dadantii and solani) analyzed in this work. **Supplementary Table 10.** Genomes of Pectobacterium carotovorum (and P. atrosepticum) analyzed in this work. **Supplementary Table 11.** ICEs identified in the genomes of the main phytopathogenic bacteria. **Supplementary Table 12.** Size and GC content of the identified elements. **Supplementary Table 13.** Attachment sites. **Supplementary Table 14.** Insertion sites, Type of Integrase and Relaxase. **Supplementary Table 15.** Putative significant cargo genes carried by ICEs characterized in this work. **Supplementary Table 16.** Elements of this work described in the literature.

**Additional file 2: Supplementary Figure 1.** Gene clusters comparision of Agrobacterium tumefaciens ICEs. **Supplementary Figure 2.** Gene clusters comparision of Dickeya ICEs. **Supplementary Figure 3.** Gene clusters comparision of Pectobacterium carotovorum (and atrosepticum) ICEs. **Supplementary Figure 4.** Gene clusters comparision of group 1 Pseudomonas syringae ICEs. **Supplementary Figure 5.** Gene clusters comparision of group 2 Pseudomonas syringae ICEs. **Supplementary Figure 6.** Gene clusters comparision of group 3 Pseudomonas syringae ICEs. **Supplementary Figure 7.** Gene clusters comparision of RSSC ICEs. **Supplementary Figure 9.** Gene clusters comparision of Xylella fastidiosa ICEs. **Supplementary Figure 10.** ICEs identity matrix heatmaps.

## Data Availability

The datasets analyzed for this study can be found in the article/Supplementary Material. The ICEs sequences here described were deposited in the ICEberg database.
