# Supplementary material for: Genomic analysis reveals the role of integrative and conjugative elements in plant pathogenic bacteria
Source: Mob DNA. 2022 Aug 12;13:19. doi: 10.1186/s13100-022-00275-1 (PMC9373382; doi:10.1186/s13100-022-00275-1)
Supplement: Supplementary file 1 — Additional file 1: Supplementary Table 1. Genomes of Pseudomonas syringae analyzed in this work. Supplementary Table 2. Genomes of Ralstonia solanacearum Species complex analyzed in this work. Supplementary Table 3. Genomes of Agrobacterium tumefaciens analyzed in this work. Supplementary Table 4. Genomes of Xanhtomonas oryzae pv. oryzae analyzed in this work. Supplementary Table 5. Genomes of Xanthomonas campestris analyzed in this work. Supplementary Table 6. Genomes of Xanthomonas axonopodis analyzed in this work. Supplementary Table 7. Genomes of Erwinia amylovora analyzed in this work. Supplementary Table 8. Genomes of Xylella fastidiosa analyzed in this work. Supplementary Table 9. Genomes of Dickeya (dadantii and solani) analyzed in this work. Supplementary Table 10. Genomes of Pectobacterium carotovorum (and P. atrosepticum) analyzed in this work. Supplementary Table 11. ICEs identified in the genomes of the main phytopathogenic bacteria. Supplementary Table 12. Size and GC content of the identified elements. Supplementary Table 13. Attachment sites. Supplementary Table 14. Insertion sites, Type of Integrase and Relaxase. Supplementary Table 15. Putative significant cargo genes carried by ICEs characterized in this work. Supplementary Table 16. Elements of this work described in the literature. [file 13100_2022_275_MOESM1_ESM.pdf]

Supplementary table 1: Genomes of *Pseudomonas syringae* analyzed in this work

Pseudomonas syringae Pathovars				
**Strain**	**RefSeq sequence**	**Isolation/Host**	**Localization**	**Pathovar**
B728a	NC_007005.1	leaflet	USA	syringae
Shaanxi_M228	NZ_CP032631.1	Actinidia	China	actinidiae
NZ-45 (ICMP 20586)	NZ_CP017007.1	*Actinidia deliciosa*	New Zealand	actinidiae
MAFF212063	NZ_CP024712.1	*Actinidia chinensis*	Japan	actinidiae
ICMP 18708	NZ_CP012179.1	Actinidia	New Zealand	actinidiae
ICMP 18884	NZ_CP011972.2	*Actinidia deliciosa*	New Zealand	actinidiae
CRAFRU14.08	NZ_CP019732.1	*Actinidia deliciosa*	Portugal	actinidiae
NZ-47	NZ_CP017009.1	*Actinidia chinensis*	New Zealand	actinidiae
CRAFRU12.29	NZ_CP019730.1	*Actinidia deliciosa*	Italy	actinidiae
P155/P220	NZ_CP032871.1	kiwifruit	China	actinidiae
ICMP 9853	NZ_CP018202.1	Actinidia	Japan	actinidiae
inb918	NZ_CP024646.1	soil	Philippines	Unknown
CFBP3846	NZ_LT963402.1	*Prunus avium*	France	avii
B301D	NZ_CP005969.1	*Pyrus communis*	United Kingdom	syringae B301D
LMG5095	NZ_CP028490.1	*Triticum aestivum*	New Zealand	atrofaciens
CFBP4215	NZ_LT962480.1	Unknown	Unknown	syringae
CFBP2116	NZ_LT985192.1	Unknown	Unknown	Unknown
CFBP2118	NZ_LT962481.1	Unknown	Unknown	syringae
CFBP6109	NZ_LT963391.1	*Prunus yedoensis*	Japan	cerasicola
CFBP3840	NZ_LT963409.1	Unknown	Unknown	Unknown
HS191	NZ_CP006256.1	*Panicum miliaceum*	Australia	syringae HS191
Pss9097	NZ_CP026568.1	Prunus sp.	United Kingdom	syringae
ATCC 10859	NZ_CP013183.1	Triticum aestivum	China	lapsa
PP1	NZ_CP034078.1	Pisum sativum	Japan	pisi
UMAF0158	NZ_CP005970.1	Mango tree	Unknown	syringae
CC1557	NZ_CP007014.1	snow	France	Unknown

UB303	NZ_CP047267.1	lake water	France	Unknown
USA011	NZ_CP045799.1	stream water	USA	Unknown

.

Supplementary table 2: Genomes of *Ralstonia solanacearum* Species complex analyzed in this work.

Ralstonia solacearum Species Complex			
**Strain**	**RefSeq sequence**	**Isolation/Host**	**Localization**
OE1-1	NZ_CP009764.1	*Solanum melongena*	Japan
FJAT-1458	NZ_CP016554.1	tomato	China
FJAT15252.F50	NZ_CP052102.1	tomato	China
FJAT1458.F1	NZ_CP052120.1	tomato	China
FJAT1458.F50	NZ_CP052118.1	tomato	China
FJAT1463.F50	NZ_CP052114.1	tomato	China
FJAT1463.F1	NZ_CP052116.1	tomato	China
FJAT15249.F50	NZ_CP052106.1	tomato	China
FJAT15252.F1	NZ_CP052104.1	tomato	China
FJAT15249.F1	NZ_CP052108.1	tomato	China
T78	NZ_CP022765.1	*Solanum tuberosum*	South Korea
EP1	NZ_CP015115.1	eggplant	China
T60	NZ_CP022768.1	*Solanum tuberosum*	South Korea
FJAT454.F1	NZ_CP052070.1	tomato	China
FJAT448.F1	NZ_CP052074.1	tomato	China
FJAT448.F50	NZ_CP052072.1	tomato	China
SL3882	NZ_CP022778.1	*Solanum tuberosum*	South Korea
FJAT454.F50-1	NZ_CP060701.1	tomato	China
SEPPX05	NZ_CP060701.1	sesame seedling	China
HA4-1	NZ_CP022481.1	peanut	China
FJAT91.F50	NZ_CP052068.1	tomato	China
FJAT91-F1	NZ_CP056083.1	Unknown	China

FJAT-91	NZ_CP016612.1	healthy tomato plant	China
FJAT91-F8	NZ_CP056085.1	Unknown	China
SL3300	NZ_CP022786.1	*Solanum tuberosum*	South Korea
FJAT15353.F8	NZ_CP052084.1	tomato	China
FJAT15353.F50	NZ_CP052086.1	tomato	China
FJAT15353.F1	NZ_CP052088.1	tomato	China
FJAT1303.F50	NZ_CP052126.1	tomato	China
FJAT1303.F8	NZ_CP052130.1	tomato	China
YC40-M	NZ_CP015850.1	Rhizoma kaempferiae	China
SL3822	NZ_CP022780.1	*Solanum tuberosum*	South Korea
CQPS-1	NZ_CP016914.1	Tobacco	China
B2	NZ_CP049787.1	tobacco substrate	China
204	NZ_CP049793.1	tobacco substrate	China
203	NZ_CP049791.1	tobacco substrate	China
202	NZ_CP049789.1	tobacco substrate	China
FJAT15244.F50	NZ_CP052110.1	tobacco substrate	China
RSCM	NZ_CP025985.1	*Cucurbita maxima*	China
FJAT15244.F1	NZ_CP052112.1	tomato	China
FJAT15244-F8	NZ_CP059376.1	tomato	China
SL3755	NZ_CP022782.1	*Solanum tuberosum*	South Korea
YQ	NZ_CP059489.1	*Casuarina equisetifolia*	China
T117	NZ_CP022755.1	*Solanum tuberosum*	South Korea
T42	NZ_CP022772.1	*Solanum tuberosum*	South Korea
GMI1000	NZ_CP022772.1F	*Solanum lycopersicum*	Unknown
SL3730	NZ_CP022784.1	*Solanum tuberosum*	South Korea
SL2729	NZ_CP022792.1	*Solanum tuberosum*	South Korea
FJAT442.F50	NZ_CP052080.1	tomato	China
FJAT442.F1	NZ_CP052082.1	tomato	China
FJAT1452.F50	NZ_CP052122.1	tomato	China

FJAT1452.F1	NZ_CP052124.1	tomato	China
FJAT445.F50	NZ_CP052076.1	tomato	China
FJAT445.F1	NZ_CP052078.1	tomato	China
SL2330	NZ_CP022794.1	*Solanum tuberosum*	South Korea
FJAT15304.F6	NZ_CP052096.1	tomato	China
FJAT15340.F50	NZ_CP052092.1	tomato	China
FJAT15304.F50	NZ_CP052098.1	tomato	China
FJAT15340.F1	NZ_CP052094.1	tomato	China
FJAT15304.F1	NZ_CP052100.1	tomato	China
FJAT15340.F6	NZ_CP052090.1	tomato	China
UW386	NZ_CP039339.1	soil	Nigeria
FJAT1303.F1	NZ_CP052128.1	tomato	China
T95	NZ_CP022761.1	*Solanum tuberosum*	South Korea
SL2064	NZ_CP022798.1	*Solanum tuberosum*	South Korea
KACC 10722	NZ_CP014702.1	*Solanum tuberosum*	South Korea
SL3022	NZ_CP023016.1	*Solanum tuberosum*	South Korea
SL3175	NZ_CP022788.1	*Solanum tuberosum*	South Korea
T98	NZ_CP022759.1	*Solanum tuberosum*	South Korea
T11	NZ_CP022776.1	*Solanum tuberosum*	South Korea
SL2312	NZ_CP022796.1	*Solanum tuberosum*	South Korea
T82	NZ_CP022763.1	*Solanum tuberosum*	South Korea
T101	NZ_CP022757.1	*Solanum tuberosum*	South Korea
T12	NZ_CP022774.1	*Solanum tuberosum*	South Korea
PSI07	NC_014311.1	Unknown	Unknown
T51	NZ_CP022770.1	*Solanum tuberosum*	South Korea
UW163	NZ_CP012939.1	plantain	Peru
SL3103	NZ_CP022790.1	*Solanum tuberosum*	South Korea
CIAT_078	NZ_CP051296.1	plantain	Colombia
Po82	NC_017574.1	Unknown	Unknown

IBSBF 2571	NZ_CP026307.1	*Musa sp.*	Brazil
KACC10709	NZ_CP016904.1	*Lycopersicon esculentum*	Korea
IBSBF1503	NZ_CP012943.1	*Cucumis sativus*	Brazil
UY031	NZ_CP012687.1	*Solanum commersonii*	Uruguay
RS 488	NZ_CP021652.1	*Solanum lycopersicum*	Brazil
RS 489	NZ_CP021766.1	*Solanum lycopersicum*	Brazil
UA-1617	NZ_CP034199.1	Plantain	Colombia
UA-1591	NZ_CP034195.1	Plantain	Colombia
UA-1611	NZ_CP034196.1	Banana	Colombia
UA-1579	NZ_CP034194.1	Banana	Colombia
UA-1609	NZ_CP034197.1	Banana	Colombia
UA-1612	NZ_CP034198.1	Banana	Colombia
FQY_4	NC_020799.1	Bacterial wilt Nursery	China
T523	NZ_CP022702.1	*Solanum lycopersicum*	Philippines
CMR15	NC_017559.1	Unknown	Unknown
P824	NZ_CP025741.1	*Vaccinium corymbosum*	USA
IBSBF 2570	NZ_CP026090.2	Musa sp.	Brazil
SFC	NZ_CP026092.2	Musa sp.	Brazil
Rs-09-161	NZ_CM002757.1	eggplant	India
Rs-10-244	NZ_CM002755.1	Chilli	India

Supplementary table 3: Genomes of *Agrobacterium tumefaciens* analyzed in this work.

		Agrobacterium tumefaciens		
**Strain**	**RefSeq sequence**	**Isolation/Host**	**Localization**	**Chromosome**
1D1609	NZ_CP026924.1	*Medicago sativa*	USA	I
1D1609	NZ_CP026925.1	*Medicago sativa*	USA	II
CFBP6625	NZ_CP039910.1	Unknown	Unknown	Unknown
CFBP6625	NZ_CP039911.1	Unknown	Unknown	Unknown
CFBP6623	NZ_CP039903.1	Unknown	Unknown	circular
CFBP6623	NZ_CP039904.1	Unknown	Unknown	linear
12D1	NZ_CP033031.1	Unknown	Unknown	circular
12D1	NZ_CP033032.1	Unknown	Unknown	linear
S33	NZ_CP014259.1	Unknown	Unknown	Unknown
S33	NZ_CP014260.1	Unknown	Unknown	Unknown
CFBP6624	NZ_CP039907.1	Unknown	Unknown	circular
CFBP6624	NZ_CP039908.1	Unknown	Unknown	linear
186	NZ_CP042274.1	*Juglans regia*	USA	circular
186	NZ_CP042275.1	*Juglans regia*	USA	linear
CFBP6626	NZ_CP039916.1	Unknown	Unknown	Unknown
CFBP6626	NZ_CP039917.1	Unknown	Unknown	Unknown
1D1460	NZ_CP032926.1	Rubus sp.	USA	circular
1D1460	NZ_CP032927.1	Rubus sp.	USA	linear
CFBP7129	NZ_CP039922.1	Unknown	Unknown	circular
CFBP7129	NZ_CP039923.1	Unknown	Unknown	linear
CFBP5499	NZ_CP039888.1	Unknown	Unknown	circular
CFBP5499	NZ_CP039889.1	Unknown	Unknown	linear
CFBP5877	NZ_CP039897.1	Unknown	Unknown	circular
CFBP5877	NZ_CP039898.1	Unknown	Unknown	linear
1D1108	NZ_CP032921.1	Euonymus sp.	USA	circular
1D1108	NZ_CP032922.1	Euonymus sp.	USA	linear

1D1108	NZ_CP033027.1	Unknown	Unknown	circular
A6	NZ_CP033028.1	Unknown	Unknown	linear
Ach5	NZ_CP011246.1	*Achillea ptarmica*	USA	circular
Ach5	NZ_CP011247.1	*Achillea ptarmica*	USA	linear
15955	NZ_CP032917.1	Unknown	Unknown	circular
15955	NZ_CP032918.1	Unknown	Unknown	linear
EML4	NZ_CP058525.1	Unknown	Unknown	circular
EML4	NZ_CP058526.1	Unknown	Unknown	linear

Supplementary table 4: Genomes of *Xanhtomonas oryzae* pv. oryzae analyzed in this work.

Xanthomonas oryzae pv. oryzae			
**Strain**	**RefSeq Sequence**	**Isolation/Host**	**Localization**
PXO99A	NC_010717.2	*Oryza sativa*	Unknown
ICMP3125	NZ_CP031697.1	*Oryza sativa*	India
NX0260	NZ_CP033192.1	*Oryza sativa*	Nepal
PXO145	NZ_CP013961.1	*Oryza sativa*	Philippines
CIAT	NZ_CP033194.1	*Oryza sativa*	Colombia
PXO211	NZ_CP013674.1	*Oryza sativa*	Philippines
PX079	NZ_CP031462.1	*Oryza sativa*	Philippines
PXO83	NZ_CP012947.1	*Oryza sativa*	Philippines
PX086	NZ_CP007166.1	*Oryza sativa* subsp. indica	Philippines
PX086-2	NZ_CP031463.1	*Oryza sativa*	Philippines
JW11089	NZ_CP033193.2	*Oryza sativa*	South Korea
PXO61	NZ_CP020942.1	*Oryza sativa*	Philippines
PXO61-2	NZ_CP021789.1	*Oryza sativa*	Philippines
PX0142	NZ_CP031698.1	*Oryza sativa*	Philippines
PXO61-3	NZ_CP021788.1	*Oryza sativa*	Philippines
KXO85	NZ_CP033197.1	*Oryza sativa*	South Korea

PXO236	NZ_CP013675.1	*Oryza sativa*	Philippines
PXO61-4	NZ_CP033187.3	*Oryza sativa*	Philippines
XF89b	NZ_CP011532.1	*Oryza sativa*	Taiwan
IX-280	NZ_CP019226.1	*Oryza sativa*	India
AUST2013	NZ_CP033196.1	*Oryza sativa*	Australia
PXO524	NZ_CP013677.1	*Oryza sativa*	Philippines
PXO602	NZ_CP013679.1	*Oryza sativa*	Philippines
JP01	NZ_CP031460.1	*Oryza sativa*	Japan
MAFF311018	NC_007705.1	Unknown	Unknown
PXO563	NZ_CP013678.1	*Oryza sativa*	Philippines
SK2-3	NZ_CP019515.1	*Oryza sativa*	Thailand
XM9	NZ_CP020334.1	*Oryza sativa*	Taiwan
PXO513	NZ_CP033188.1	*Oryza sativa*	Philippines
HuN37	NZ_CP031456.1	*Oryza sativa*	China
PXO404	NZ_CP033190.1	*Oryza sativa*	Philippines
PXO421	NZ_CP033189.1	*Oryza sativa*	Philippines
PXO71	NZ_CP013670.1	*Oryza sativa*	Philippines
PXO364	NZ_CP033191.1	*Oryza sativa*	Philippines
JL25	NZ_CP031457.1	*Oryza sativa*	China
JL33	NZ_CP031459.1	*Oryza sativa*	China
YC11	NZ_CP031464.1	*Oryza sativa*	China
ScYc-b	NZ_CP031469.1	*Oryza sativa*	China
ScYc-b	NZ_CP018087.1	*Oryza sativa*	China
CFBP7319	NZ_CP033181.1	*Oryza sativa*	Burkina Faso
CIX298	NZ_CP036378.1	*Oryza sativa*	Burkina Faso
CFBP7340	NZ_CP033174.1	*Oryza sativa*	Niger
mai/01	NZ_CP025609.1	*Oryza sativa*	Mali
CFBP1949	NZ_CP033184.1	*Oryza sativa*	Mali
CFBP7325	NZ_CP033176.1	*Oryza sativa*	Mali

MAI134	NZ_CP019091.1	*Oryza longistaminata*	Mali
CFBP7337	NZ_CP033175.1	*Oryza sativa*	Mali
CIX2374	NZ_CP036377.1	*Oryza sativa*	Senegal
CFBP7323	NZ_CP033178.1	*Oryza sativa*	Niger
Ug11	NZ_CP033170.1	*Oryza sativa*	Uganda
Dak16	NZ_CP033172.1	*Oryza sativa*	Tanzania
T19	NZ_CP033171.1	*Oryza sativa*	Tanzania
CFBP7324	NZ_CP033177.1	*Oryza sativa*	Niger
CFBP7320	NZ_CP033186.1	*Oryza sativa*	Burkina Faso
BAI3	NZ_CP025610.1	*Oryza sativa*	Burkina Faso
CFBP7321	NZ_CP033180.1	*Oryza sativa*	Burkina Faso
CFBP7322	NZ_CP033179.1	*Oryza sativa*	Burkina Faso
MAI106	NZ_CP019089.1	*Oryza sativa*	Mali
mai/95	NZ_CP019087.1	*Oryza sativa*	Mali
CFBP1951	NZ_CP033183.1	*Oryza sativa*	Mali
mai/73	NZ_CP019086.1	*Oryza sativa*	Mali
MAI145	NZ_CP019092.1	*Oryza sativa*	Mali
MAI129	NZ_CP019090.1	*Oryza sativa*	Mali
mai/68	NZ_CP019085.1	*Oryza sativa*	Mali
mai/99	NZ_CP019088.1	*Oryza sativa*	Mali
JL28	NZ_CP031458.1	*Oryza sativa*	China
CFBP1952	NZ_CP033182.1	*Oryza sativa*	Mali
AXO1947	NZ_CP013666.1	*Oryza sativa*	Cameroon
CFBP1948	NZ_CP033185.1	*Oryza sativa*	Cameroon
CFBP8172	NZ_CP033173.1	*Oryza sativa*	Benin
YN24	Unknown	*Oryza sativa*	China
BXO1	Unknown	*Oryza sativa*	India
PXO282	NZ_CP013676.1	*Oryza sativa*	Philippines
KACC10331	NC_006834.1	Unknown	Unknown

OS198	NZ_CP031461.1	*Oryza sativa*	China
IXO1088	NZ_CP040687.1	*Oryza sativa*	India
IXO704	NZ_CP040604.1	*Oryza sativa*	India
K2	NZ_CP050113.1	*Oryzae sativa* L.	South Korea
K1	NZ_CP049205.1	*Oryzae sativa* L.	South Korea
K3	NZ_CP050114.1	*Oryzae sativa* L.	South Korea
K3a	NZ_CP050115.1	*Oryzae sativa* L.	South Korea
ITCCBB0002	NZ_CP046148.1	*Oryza sativa*	India
LN18	Unknown	*Oryza sativa*	China

Supplementary table 5: Genomes of *Xanthomonas campestris* analyzed in this work.

***Xanthomonas campestris* pahtovars**				
**Strain**	**RefSeq Sequence**	**Isolation/Host**	**Localization**	**Pathovar**
ATCC 33913	NC_003902.1	*Brassica oleracea*	Unknown	campestris
8004	NC_007086.1	*Brassica oleracea var. botrytis*	United Kingdom	campestris
CN18	NZ_CP017319.1	*Brassica juncea var. foliosa*	China	campestris
CN17	NZ_CP017307.1	*Brassica rapa subsp. Chinensis*	China	campestris
CN12	NZ_CP017310.1	*Brassica napus subsp. Oleifera*	China	campestris
B100	NC_010688.1	Mud	Germany	campestris
3811	NZ_CP025750.1	Cabbage	Unknown	campestris
MAFF302021	NZ_AP019684.1	*Brassica oleracea var. botrytis*	Japan	campestris
CN03	NZ_CP017308.1	*Brassica rapa subsp. Pekinensis*	Japan	campestris
CN14	NZ_CP017317.1	*Brassica juncea var. foliosa*	China	campestris
CN15	NZ_CP017323.1	*Brassica rapa subsp. Chinensis*	China	campestris
MAFF106712		Unknown	Unknown	campestris
NEB122	NZ_CP051651.1	Unknown	Unknown	badrii
756C	NC_017271.1	Unknown	Unknown	raphani
ICMP 21080	NZ_CP012145.1	cabbage	New Zealand	campestris

ICMP 4013	NZ_CP012146.1	*Brassica oleracea var. capitata*	New Zealand	campestris
ICMP 4013	NZ_CP051651.1	Unknown	Unknown	badrii
MAFF106181	NZ_CP058243.1	Japanese radish	Japan	raphani

Supplementary table 6: Genomes of *Xanthomonas axonopodis* analyzed in this work.

		Xanthomonas axonopodis		
**Strain**	**RefSeq Sequence**	**Isolation/Host**	**Localization**	**Pathovar**
Xac29-1	NC_020800.1	Unknown	Unknown	Unknown
LMG26789	NZ_CP031059.1	*Commiphora wightii*	India	commiphoreae
NCPPB 796	NZ_CP053649.1	sugarcane	Mauritius	vasculorum

Supplementary table 7: Genomes of *Erwinia amylovora* analyzed in this work.

	**Erwinia amylovora**		
**Strain**	**RefSeq Sequence**	**Isolation/Host**	**Localization**
E-2	NZ_CP024970.1	Malus sp.	Belarus
CFPB1430	NC_013961.1	Unknown	Unknown
ATCC 49946	NC_013971.1	Unknown	Unknown
FB-86	NZ_CP050258.1	Apple tree	South Korea
FB-207	NZ_CP050263.1	Pear tree	South Korea
TS3238	NZ_CP050244.1	Pear tree	South Korea
TS3128	NZ_CP056034.1	*Pyrus pyrifolia var. culta*	South Korea
FB-307	NZ_CP050242.1	Apple tree	South Korea
FB-20	NZ_CP050240.1	Pear tree	South Korea

Supplementary table 8: Genomes of *Xylella fastidiosa* analyzed in this work.

	Xylella fastidiosa		
**Strain**	**RefSeq Sequence**	**Isolation/Host**	**Localization**
9a5c	NC_002488.3	Orange Valencia	Brazil
Hib4	NZ_CP009885.1	Hibiscus	Brazil
J1a12	NZ_CP009823.1	Citrus	Brazil
Ann-1	NZ_CP006696.1	Oleander	Unknown
3124	NZ_CP009829.1	coffee	Brazil
U24D	NZ_CP009790.1	*Citrus sinensis*	Brazil
Pr8x	NZ_CP009826.1	Plum	Brazil
Fb7	NZ_CP010051.2	Citrus	Argentina
MUL0034	NZ_CP006740.1	Mulberry	Unknown
M23	NC_010577.1	*Prunus dulcis*	USA
Temecula1	NC_004556.1	Grapevine	USA
De Donno	NZ_CP020870.1	*Olea europaea*	Italy
Salento-2	NZ_CP016610.1	*Olea europaea*	Italy
Salento-1	NZ_CP016608.1	*Olea europaea*	Italy
Bakersfield-1	NZ_CP040799.1	*Vitis vinifera*	USA
GB514	NC_017562.1	grape	USA
M12	NC_010513.1	*Prunus dulcis*	USA
ATCC 35879	NZ_CP044352.1	Grapevine	USA
RH1	NZ_CP052853.1	*Olea europaea*	USA
LM10	NZ_CP052854.1	*Olea europaea*	USA
Fillmore	NZ_CP052855.1	*Olea europaea*	USA

Supplementary table 9: Genomes of *Dickeya (dadantii* and *solani)* analyzed in this work.

	Dickeya dadantii		
**Strain**	**RefSeq**	**Host**	**Localization**
DSM 18020	NZ_CP023467.1	*Pelargonium capitatum*	Comoros
3937	NC_014500.1	African violet	Unknown
	Dickeya solani		
**Strain**	**RefSeq**	**Host**	**Localization**
IOP2222	NZ_CP015137.1	*Solanum tuberosum*	Netherlands
IFB0223	NZ_CP024710.1	*Solanum tuberosum*	Germany
IFB 0099	NZ_CP024711.1	*Solanum tuberosum*	Poland
RNS 08.23.3.1.A	NZ_CP016928.1	*Solanum tuberosum*	France
D s0432-1	NZ_CP017453.1	*Solanum tuberosum*	Finland
PPO 9019	NZ_CP017454.1	Muscari	Netherlands
IFB0223	NZ_CP024710.1	*Solanum tuberosum*	Germany
IFB0421	NZ_CP051460.1	*Solanum tuberosum*	Portugal
IFB0231	NZ_CP051458.1	*Solanum tuberosum*	Finland
IFB0417		*Solanum tuberosum*	Portugal
IFB0167	NZ_CP051457.1	*Solanum tuberosum*	Poland

Supplementary table 10: Genomes of *Pectobacterium carotovorum* (and *P. atrosepticum*) analyzed in this work.

	Pectobacterium carotovorum		
**Strain**	**RefSeq**	**Host**	**Localization**
PC1	NC_012917.1	Unknown	Unknown
PCC21	NC_018525.1	Unknown	Unknown
JR1.1	NZ_CP034237.1	Radish	South Korea
BP201601.1	NZ_CP034236.1	Potato	South Korea
	Pectobacterium atrosepticum		
**Strain**	**RefSeq**	**Host**	**Localization**
SCRI1043	NC_004547.2	Solanum tuberosum	Unknown
JG10-08	NZ_CP007744.1	Solanum tuberosum	China
21A	NZ_CP009125.1	Solanum tuberosum	Belarus
36A	NZ_CP024956.1	Solanum tuberosum	Belarus

Supplementary table 11: ICEs identified in the genomes of the main phytopathogenic bacteria

Strain	Element	RefSeq	Location (nt)	Length (bp)
		Dickeya dadantii		
3937	*ICEDda1*	NC_014500.1	3126494-3201103	74.610
DSM 18020	*ICEDda2*	NZ_CP023467.1	866896-926592	40.117
		Dickeya solani		
**Strain**	**Element**	**RefSeq**	**Location (nt)**	**Length (bp)**
IPO2222		NZ_CP015137.1	4033605-4081983	48.378
IFB0223		NZ_CP024710.1	1870124-1918502	48.378
IFB 0099		NZ_CP024711.1	1873274-1921652	48.378
RNS 08.23.3.1.A		NZ_CP016928.1	4694369-4742747	48.378
D s0432-1		NZ_CP017453.1	835683-884061	48.378
PPO 9019	*ICEDso1*	NZ_CP017454.1	828904-877282	48.378
IFB0421		NZ_CP051460.1	1873239-1921616	48.377
IFB0231		NZ_CP051458.1	1873279-1921657	48.378
IFB0417		CP051459.1	1872976-1921336	48.360
IFB0223		NZ_CP024710.1	1870124-1918502	48.378
IFB0167		NZ_CP051457.1	1873279-1921657	48.378
		Pectobacterium atrosepticum		
**Strain**	**Element**	**RefSeq**	**Location (nt)**	**Length (bp)**
SCRI1043	*ICEPca1*	NC_004547.2	590755-688599	97.845
SCRI1043	*ICEPca2*		1867875-1926959	59.085
JG10-08	*ICEPca3*	NZ_CP007744.1	3165718-3228840	63.123
JG10-08			980430-1077384	96.954
21A	*ICEPca1*	NZ_CP009125.1	975442 -1072594	97.152
36A		NZ_CP024956.1	586814-683968	97.154

Strain	Element	RefSeq	Location (nt)	Length (bp)
36A	*ICEPca4*		1863011-1908923	45.912
Pectobacterium carotovorum				
**Strain**	**Element**	**RefSeq**	**Location (nt)**	**Length (bp)**
JR1.1	*ICEPcc1*	NZ_CP034237.1	1595656-1632307	36.651
BP201601.1	*ICEPcc2*	NZ_CP034236.1	1663873-1730176	66.303
Agrobacterium tumefaciens				
**Strain**	**Element**	**RefSeq**	**Location (nt)**	**Length (bp)**
1D1609 (Cromossomo I)	*ICEAtu1*	NZ_CP026924.1	272270-334336	61.887
12D1 (Cromossomo circular)	*ICEAtu2*	NZ_CP033031.1	2309372-2377994	68.662
12D1 (Cromossomo linear)	*ICEAtu3*	NZ_CP033032.1	1340555-1455519	114.964
186(Cromossomo circular)	*ICEAtu4*	NZ_CP042274.1	2013312-2114889	101.577
Pseudomonas syringae				
**Strain**	**Element**	**RefSeq**	**Location (nt)**	**Length (bp)**
B728a	*ICEPsy1*	NC_007005.1	1604658-1724374	119.716
Shaanxi_M228	*ICEPsy2*	NZ_CP032631.1	5463863-5614284	150.421
Shaanxi_M228	*ICEPsy3*		1672240-1753644	81.404
Shaanxi_M228	*ICEPsy4*		1753496-1862291	108.795
Shaanxi_M228	*ICEPsy5*		1862882-1911082	48.201
NZ-45 (ICMP 20586)	*ICEPsy2*	NZ_CP017007.1	5369000-5512238	143.238
NZ-45 (ICMP 20586)	*ICEPsy6*		1734480-1836698	102.218
NZ-45 (ICMP 20586)	*ICEPsy7*		1835321-1892784	57.463
NZ-45 (ICMP 20586)	*ICEPsy8*		5513439-5621120	107.200
MAFF212063	*ICEPsy9*	NZ_CP024712.1	4717464-4816692	99.228
MAFF212063	*ICEPsy10*		5548512-5709520	161.009
ICMP 18708	*ICEPsy2*	NZ_CP012179.1	5266321-5409559	143.238
ICMP 18708	*ICEPsy7*		1734473-1791958	57.485
ICMP 18884	*ICEPsy2*	NZ_CP011972.2	5266318-5409556	143.238
ICMP 18884	*ICEPsy7*		1734470-1784283	49.813

CRAFRU14.08	*ICEPsy2*		1829573-1980910	151.337
CRAFRU14.08	*ICEPsy11*	NZ_CP019732.1	1735800-1828308	92.508
CRAFRU14.08	*ICEPsy12*		5462941-5504692	41.751
NZ-47	*ICEPsy2*		5266318-5409556	143.238
NZ-47	*ICEPsy7*	NZ_CP017009.1	1734470-1784322	49.852
NZ-47	*ICEPsy13*		5410536-5501754	91.218
CRAFRU12.29	*ICEPsy2*		5360196-5503434	143.238
CRAFRU12.29	*ICEPsy11*	NZ_CP019730.1	1737471-1829979	92.508
CRAFRU12.29	*ICEPsy7*		1830465-1880317	49.852
P155/P220	*ICEPsy14*	NZ_CP032871.1	1689937-1797268	107.331
P155/P220	*ICEPsy2*		5384241-5527881	143.640
CFBP6109	*ICEPsy15*	NZ_LT963391.1	1050946-1102190	51.244
CFBP3840	*ICEPsy16*	NZ_LT963409.1	4987773-5066398	78.625
HS191	*ICEPsy17*	NZ_CP006256.1	5140209-5226281	85.921
PP1	*ICEPsy18*	NZ_CP034078.1	865150-953883	89.125
CC1557	*ICEPsy19*	NZ_CP007014.1	2591336-2746640	155.304
UB303	*ICEPsy20*	NZ_CP047267.1	5453047-5484042	30.968
		Xanthomonas campestris		
**Strain**	**Element**	**RefSeq**	**Location (nt)**	**Length (bp)**
B100	*ICEXca1*	NC_010688.1	2798361-2862467	64.106
3811		NZ_CP025750.1	2798845-2863378	64.534
CN03	*ICEXca2*	NZ_CP017308.1	2398353-2482109	83.757
		Xylella fastidiosa		
**Strain**	**Element**	**RefSeq**	**Location (nt)**	**Length (bp)**
9a5c	*ICEXfa1*	NC_002488.3	1934170-2022372	88.202
Hib4	*ICEXfa2*	NZ_CP009885.1	1399531-1497444	97.913
J1a12	*ICEXfa3*	NZ_CP009823.1	1931410-2069541	158.355
U24D	*ICEXfa1*	NZ_CP009790.1	1934275 - 2022404	88.129

R. pseudosolanacearum				
**Strain**	**Element**	**RefSeq**	**Location (nt)**	**Length (bp)**
GMI1000	*Tn4371*	NC_003295.1	2780151-2825764	45.613
RS476		NZ_CP021762.1	2780170-2825773	45.603
CRMRs218		NZ_CP021764.1	2780419-2826032	45.613
FJAT-91	*ICERps1*	NZ_CP016612.1	1283877-1335170	51.293
FQY_4	*ICERps2*	NC_020799.1	1113417-1173796	60.379
YC40-M		NZ_CP015850.1	2532466-2588165	55.699
HA4I	*ICERps3*	NZ_CP022481.1	1709954-1756828	46.874
UW386	*ICERps4*	NZ_CP039339.1	545761-602147	56.386
FJAT91-F1	*ICERps1*	NZ_CP056083.1	2505206-2556182	50.976
FJAT91-F8		NZ_CP056085.1	2505097-2556073	50.976

R. syzyzii				
**Strain**	**Element**	**RefSeq**	**Location (nt)**	**Length (bp)**
T98	*ICERsy1*	NZ_CP022759.1	1925451-1985747	60.296
SL3175		NZ_CP022788.1	1925467-1985763	60.296

Supplementary table 12: Size and GC content of the identified elements

Strain	Element	Location (nt)	RefSeq	Genome Length (bp)	Element Length (bp)	Genome %GC	Element %GC
Dickeya dadantii							
3937	*ICEDda1*	3126494-3201103	NC_014500.1	4.922.802	74.610	56.3	52.1
DSM 18020	*ICEDda2*	876895-917012	NZ_CP023467.1	4.997.541	40.117	56.4	52.9
Dickeya solani							
**Strain**	**Element**	**Location (nt)**	**RefSeq**	**Genome Length (bp)**	**Element Length (bp)**	**Genome %GC**	**Element %GC**
IOP2222		4033605-4081983	NZ_CP015137.1	4.919.833			
IFB0223		1870124-1918502	NZ_CP024710.1	4.937.554		56.20	
IFB 0099		1873274-1921652	NZ_CP024711.1	4.932.920	48.378		
RNS 08.23.3.1.A		4694369-4742747	NZ_CP016928.1	4.922.468		56.30	
D s0432-1		835683-884061	NZ_CP017453.1	4.919.812		56.20	
PPO 9019	*ICEDso1*	828904-877282	NZ_CP017454.1	4.918.850		56.29	49.4
IFB0421		1873239-1921616	NZ_CP051460.1	4.934.537	48.377		
IFB0231		1873279-1921657	NZ_CP051458.1	4.924.702	48.378		
IFB0417		1872976-1921336	CP051459.1	4.924.102	48.360	56.20	
IFB0223		1870124-1918502	NZ_CP024710.1	4.937.554	48.378		
IFB0167		1873279-1921657	NZ_CP051457.1	4.922.289			
Pectobacterium atrosepticum							
**Strain**	**Element**	**Location (nt)**	**RefSeq**	**Genome Length (bp)**	**Element Length (bp)**	**Genome %GC**	**Element %GC**
SCRI1043	*ICEPca1*	590755-688599	NC_004547.2	5.064.019	97.845	51.00	48.3
SCRI1043	*ICEPca2*	1867875-1926959		5.064.019	59.085		40.7

JG10-08	*ICEPca3*	3165718-3228840	NZ_CP007744.1	5.004.926	63.123	51.10	48.3
JG10-08		980430-1077384		5.004.926	96.954		48.3
21A	*ICEPca1*	975442 -1072594	NZ_CP009125.1	4.991.806	97.152	51.07	48.3
36A		586814-683968	NZ_CP024956.1	4.965.575	97.154	51.10	48.3
36A	*ICEPca4*	1863011-1908923		4.965.575	45.912		46.9

Pectobacterium carotovorum

**Strain**	**Element**	**Location (nt)**	**RefSeq**	**Genome Length (bp)**	**Element Length (bp)**	**Genome %GC**	**Element %GC**
JR1.1	*ICEPcc1*	1595656-1632307	NZ_CP034237.1	4.872.902	36.651	52.00	45.5
BP201601.1	*ICEPcc2*	1663873-1730176	NZ_CP034236.1	4.853.176	66.303	52.20	51.3

Agrobacterium tumefaciens

**Strain**	**Element**	**Location (nt)**	**RefSeq**	**Genome Length (bp)**	**Element Length (bp)**	**Genome %GC**	**Element %GC**
1D1609 (Cromossomo I)	*ICEAtu1*	272270-334336	NZ_CP026924.1	3.058.772	61.887	59.49	63.7
12D1 (Cromossomo circular)	*ICEAtu2*	2309372-2377994	NZ_CP033031.1	3.027.766	68.662	59.53	58.7
12D1 (Cromossomo linear)	*ICEAtu3*	1340555-1455519	NZ_CP033032.1	2.258.260	114.964		58.1
186(Cromossomo circular)	*ICEAtu4*	2013312-2114889	NZ_CP042274.1	2.943.916	101.577	59.43	61.0

Pseudomonas syringae

**Strain**	**Element**	**Location (nt)**	**RefSeq**	**Genome Length (bp)**	**Element Length (bp)**	**Genome %GC**	**Element %GC**
B728a	*ICEPsy1*	1604658-1724374	NC_007005.1	6.093.698	119.716	58.70	54.5
Shaanxi_M228	*ICEPsy2*	5463863-5614284	NZ_CP032631.1	6.674.594	150.421	58.28	57.0
Shaanxi_M228	*ICEPsy3*	1672240-1753644		6.674.594	81.404		54.3
Shaanxi_M228	*ICEPsy4*	1753496-1862291		6.674.594	108.795		54.3
Shaanxi_M228	*ICEPsy5*	1862882-1911082		6.674.594	48.201		54.4
NZ-45 (ICMP 20586)	*ICEPsy2*	5369000-5512238	NZ_CP017007.1	6.665.031	143.238	58.37	57.2
NZ-45 (ICMP 20586)	*ICEPsy6*	1734480-1836698		6.665.031	102.218		54.8
NZ-45 (ICMP 20586)	*ICEPsy7*	1835321-1892784		6.665.031	57.463		53.9
NZ-45 (ICMP 20586)	*ICEPsy8*	5513439-5621120		6.665.031	107.200		55.2

MAFF212063	*ICEPsy9*	4717464-4816692	NZ_CP024712.1	6.556.999	99.228	58.35	54.7
MAFF212063	*ICEPsy10*	5548512-5709520		6.556.999	161.009		55.3
ICMP 18708	*ICEPsy2*	5266321-5409559	NZ_CP012179.1	6.555.571	143.238	58.60	57.2
ICMP 18708	*ICEPsy7*	1734473-1791958		6.555.571	57.485		53.9
ICMP 18884	*ICEPsy2*	5266318-5409556	NZ_CP011972.2	6.555.569	143.238		57.2
ICMP 18884	*ICEPsy7*	1734470-1784283		6.555.569	49.813		53.9
CRAFRU14.08	*ICEPsy2*	1829573-1980910		6.546.028	151.337	58.37	57.2
CRAFRU14.08	*ICEPsy11*	1735800-1828308	NZ_CP019732.1	6.546.028	92.508		54.4
CRAFRU14.08	*ICEPsy12*	5462941-5504692		6.546.028	41.751		53.7
NZ-47	*ICEPsy2*	5266318-5409556		6.545.910	143.238		57.2
NZ-47	*ICEPsy7*	1734470-1784322	NZ_CP017009.1	6.545.910	49.852	58.40	53.9
NZ-47	*ICEPsy13*	5410536-5501754		6.545.910	91.218		55.1
CRAFRU12.29	*ICEPsy2*	5360196-5503434		6.545.549	143.238		57.2
CRAFRU12.29	*ICEPsy11*	1737471-1829979	NZ_CP019730.1	6.545.549	92.508		54.4
CRAFRU12.29	*ICEPsy7*	1830465-1880317		6.545.549	49.852	58.37	53.9
P155/P220	*ICEPsy14*	1689937-1797268	NZ_CP032871.1	6.529.859	107.331		54.8
P155/P220	*ICEPsy2*	5384241-5527881		6.529.859	143.640		57.2
CFBP6109	*ICEPsy15*	1050946-1102190	NZ_LT963391.1	6.015.874	51.244	58.10	55.9
CFBP3840	*ICEPsy16*	4987773-5066398	NZ_LT963409.1	6.013.125	78.625	58.06	54.8
HS191	*ICEPsy17*	5140209-5226281	NZ_CP006256.1	5.950.211	86.072	58.96	54.6
PP1	*ICEPsy18*	865150-953883	NZ_CP034078.1	5.883.416	88.734	58.80	54.6
CC1557	*ICEPsy19*	2591336-2746640	NZ_CP007014.1	5.758.024	155.304	58.56	57.0
UB303	*ICEPsy20*	5453047-5484042	NZ_CP047267.1	6.141.482	30.968	59.20	50.7

Xanthomonas campestris

**Strain**	**Element**	**Location (nt)**	**RefSeq**	**Genome Length (bp)**	**Element Length (bp)**	**Genome GC**	**Element GC**
B100	*ICEXca1*	2798361-2862467	NC_010688.1	5.079.002	64.106	65.00	61.7
3811		2798845-2863378	NZ_CP025750.1	5.072.566	64.534		

CN03	*ICEXca2*	2398353-2482109	NZ_CP017308.1	5.035.984	83.756	65.05	60.1

Xylella fastidiosa

**Strain**	**Element**	**Location (nt)**	**RefSeq**	**Genome Length (bp)**	**Element Length (bp)**	**Genome GC**	**Element GC**
9a5c	*ICEXfa1*	1934170-2022372	NC_002488.3	2.813.297	88.202	52.64	52.9
Hib4	*ICEXfa2*	1399531-1497444	NZ_CP009885.1	2.813.297	97.913	52.69	66.4
J1a12	*ICEXfa3*	1931410-2069541	NZ_CP009823.1	2.788.789	158.355	52.81	54.6
U24D	*ICEXfa1*	1934275-2022404	NZ_CP009790.1	2.681.334	88.129	52.64	53

Ralstonia pseudosolanacearum

**Strain**	**Element**	**Location (nt)**	**RefSeq**	**Genome Length (bp)**	**Element Length (bp)**	**Genome GC**	**Element GC**
GMI1000		2780151-2825764	NC_003295.1	3.716.413	45.613	66.96	63.2
RS476	*Tn4371*	2780170-2825773	NZ_CP021762.1	3.716.422	45.603	66.96	63.2
CRMRs218		2780419-2826032	NZ_CP021764.1	3.716.755	45.613	66.70	63.2
FJAT-91	*ICERps1*	1283877-1335170	NZ_CP016612.1	3.873.214	51.293	66.87	60.9
FQY_4	*ICERps2*	1113417-1173796	NC_020799.1	3.715.422	60.379	66.79	63
YC40-M		2532466-2588165	NZ_CP015850.1	3.844.764	55.699	66.70	62.3
HA4I	*ICERps3*	1709954-1756828	NZ_CP022481.1	3.890.347	46.874	66.68	62.2
UW386	*ICERps4*	545761-602147	NZ_CP039339.1	3.658.282	56.386	66.93	62.4
FJAT91-F1	*ICERps1*	2505206-2556182	NZ_CP056083.1	3.873.221	50.976	66.87	60.9
FJAT91-F8		2505097-2556073	NZ_CP056085.1	3.873.088	50.976	66.87	60.9

Ralstonia syzyzii

**Strain**	**Element**	**Location (nt)**	**RefSeq**	**Genome Length (bp)**	**Element Length (bp)**	**Genome GC**	**Element GC**
T98	*ICERsy1*	1925451-1985747	NZ_CP022759.1	3.569.696	60.296	66.33	60.2
SL3175		1925467-1985763	NZ_CP022788.1	3.569.709	60.296	66.33	60.2

Supplementary table 13: Attachment sites

Element	Strain	Sequence	AttL	AttR
		Dickeya dadantii		
**Element**	**Strain**	**Sequence**	**AttL**	**AttR**
ICEDda1	3937	ccagtcagaggagccaaa	3126494-3126511	3201086-3201103
		Dickeya solani		
**Element**	**Strain**	**Sequence**	**AttL**	**AttR**
ICEDso1	IPO2222	tggctcctctgactgg	4033605-4033620	4081968-4081983
ICEDso1	IFB0223	tggctcctctgactgg	1870124-1870139	1918487-1918502
ICEDso1	IFB 0099	tggctcctctgactgg	1873274-1873289	1921637-1921652
ICEDso1	RNS 08.23.3.1.A	tggctcctctgactgg	4694369-4694384	4742732-4742747
ICEDso1	D s0432-1	ccagtcagaggagcca	835683-835698	884046-884061
ICEDso1	PPO 9019	ccagtcagaggagcca	828904-828919	877267-877282
ICEDso1	IFB0421	tggctcctctgactgg	1873239-1873254	1921601-1921616
ICEDso1	IFB0231	tggctcctctgactgg	1873279-1873294	1921642-1921657
ICEDso1	IFB0417	tggctcctctgactgg	1872976-1872991	1921321-1921336
ICEDso1	IFB0223	tggctcctctgactgg	1870124-1870139	1918487-1918502
ICEDso1	IFB0167	tggctcctctgactgg	1873279-1873294	1921642-1921657
		Pectobacterium atrosepticum		
**Element**	**Strain**	**Sequence**	**AttL**	**AttR**
ICEPca1	SCRI1043	agtggtgcccggactcgg	590755-590803	688582-688599
ICEPca2	SCRI1043	ttggctcctctgactggactcgaa	1886342-1886365	1927138-1927161
ICEPca3	JG10-08	atcgggagaatttggtggtcagatttggggtca	3165718-3165750	3228808-3228840
ICEPca1	JG10-08	agtggtgcccggactcgg	980430-980447	1077564-1077581
ICEPca1	21A	agtggtgcccggactcgg	975442-975459	1072576-1072593
ICEPca1	36A	agtggtgcccggactcgga	586814-586862	683950-683968
ICEPca4	36A	aatttggctcctctgactgg	1863011-1863030	1908904-1908923

		Pectobacterium carotovorum		
**Element**	**Strain**	**Sequence**	**AttL**	**AttR**
ICEPcc1	JR1.1	aatttggctcctctgactgg	1595656-1595675	1632288-1632307
ICEPcc2	BP201601.1	atttgggggtcactccgtcatcgaaccaa	1663873-1663901	1730148-1730176
		Agrobacterium tumefaciens		
**Element**	**Strain**	**Sequence**	**AttL**	**AttR**
ICEAtu3	12D1 (Cromossomo linear)	gcccccgcaaccaccga	1340555-1340571	1455503-1455519
ICEAtu4	186(Cromossomo circular)	gctccttcagcaccg	2015364-2015378	2114875-2114889
		Pseudomonas syringae		
**Element**	**Strain**	**Sequence**	**AttL**	**AttR**
ICEPsy1	B728a	acaagcagccgtctg	1604658-1604672	1724360-1724374
ICEPsy2	Shaanxi_M228	agttttccggccatt	5463863-5463877	5614270-5614284
ICEPsy3	Shaanxi_M228	ttggcttttaaccaattggtcgtaggttcgaatcccacacgacccaccattt	1672243-1672294	1753593-1753644
ICEPsy4	Shaanxi_M228	tataaagcctctaatggggacattagag	1753503-1753530	1862258-1862285
ICEPsy2	NZ-45 (ICMP 20586)	agttttccggccatt	5369000-5369014	5512224-5512238
ICEPsy6	NZ-45 (ICMP 20586)	ctgacgtaggattttgtggcagcccaatagatctt	1735777- 1735811	1836664-1836698
ICEPsy8	NZ-45 (ICMP 20586)	tgcgacctgatacgagtggcatcagatctttaagattcggctccaaggt	5513439-5513487	5621072-5621120
ICEPsy9	MAFF212063	aaatggtgggtcgtgtgggattcgaacctacgaccaattggttaaaag	4717464-47175511	4816645-4816692
ICEPsy2	ICMP 18708	agttttccggccatt	5266321-5266335	5409545-5409559
ICEPsy2	ICMP 18884	agttttccggccatt	5266318-5266332	5409542-5409556
ICEPsy2	CRAFRU14.08	aatggccggaaaact	1829573-1829587	1980896-1980910
ICEPsy2	NZ-47	agttttccggccatt	5266318-5266332	5409542-5409556
*ICEPsy*13	NZ-47	gcgttatgtttcgcgacagacggctgcttg	5410536-5410565	5501725-5501754
ICEPsy2	CRAFRU12.29	agttttccggccatt	5360196-5360210	5503420-5503434
*ICEPsy*11	CRAFRU12.29	ttggcttttaaccaattggtcgtaggttcgaatcccacacgacccaccattt	1737471-1737522	1829928-1829979
*ICEPsy*14	P155/P220	ttggcttttaaccaattggtcgtaggttcgaatcccacacgacccaccattt	1689937-1689988	1797217-1797268

ICEPsy2	P155/P220	agttttccggccatt	5384241-5384255	5527867-5527881
*ICEPsy*16	CFBP3840	gtggcatcagatctttaagattcggctccaaggtgaaccttggaaccaaaaatggtgggtcgtgtgggattcgaacctacgaccaattggttaaaag	4987773-7987869	50663302-5066398
ICEPsy17	HS191	tggtcggggtaaggggattcgaactcctgacatcctgctcccaaagcaggcgcgctaccggactgcgctataccccg	5140209-5140285	5226205-5226281
*ICEPsy*18	PP1	cggggtatagcgcagtccggtagcgcgcctgctttgggagcaggatgtcaggagttcgaatccccttaccccgacca	864759-864835	953807-953883
*ICEPsy*19	CC1557	gtctcgtttcccgctcca	2591336-2591353	2746623-2746640
*ICEPsy*20	UB303	actcataatcctttggtccacggttcgagtccgtgtgggcccacca	5453003-5453048	5484451-5484496
		Xanthomonas campestris		
**Element**	**Strain**	**Sequence**	**AttL**	**AttR**
ICEXca1	B100	agcgagcgccgggcttg	2798361-2798377	2862451-2862467
ICEXca1	3811	agcgagcgccgggcttg	2798845-2798861	2863362-2863378
ICEXca2	CN03	gagggtcggcagggaatcattac	2398353-2398375	2482087-2482109
		Xylella fastidiosa		
**Element**	**Strain**	**Sequence**	**AttL**	**AttR**
ICEXfa2	Hib4	ctcgtttcccgctcca	1399531-1399546	1497429-1497444

Supplementary table 14: Insertion sites, Type of Integrase and Relaxase

Strain	Element	RefSeq	Insertion site	Integrase	Relaxase
Dickeya dadantii					
3937	*ICEDda1*	NC_014500.1	tRNA-Asn	Phage	MOBC
DSM 18020	*ICEDda2*	NZ_CP023467.1	BamE	Tyr	MOBP
Dickeya solani					
**Strain**	**Element**	**RefSeq**	**Insertion site**	**Integrase**	**Relaxase**
IOP2222	*ICEDso1*	NZ_CP015137.1	tRNA-Asn	Tyr	MOBC
IFB0223	*ICEDso1*	NZ_CP024710.1			
IFB 0099	*ICEDso1*	NZ_CP024711.1			
RNS 08.23.3.1.A	*ICEDso1*	NZ_CP016928.1			
D s0432-1	*ICEDso1*	NZ_CP017453.1			
PPO 9019	*ICEDso1*	NZ_CP017454.1			
IFB0223	*ICEDso1*	NZ_CP024710.1			
IFB0421	*ICEDso1*	NZ_CP051460.1			
IFB0231	*ICEDso1*	NZ_CP051458.1			
IFB0223	*ICEDso1*	CP051459.1			
IFB0417	*ICEDso1*	NZ_CP024710.1			
IFB0167	*ICEDso1*	NZ_CP051457.1			
Pectobacterium atrosepticum					
**Strain**	**Element**	**RefSeq**	**Insertion site**	**Integrase**	**Relaxase**
SCRI1043	*ICEPca1*	NC_004547.2	tRNA-Phe	Tyr	TraI
SCRI1043	*ICEPca2*		tRNA-Asn	Phage	MOBQ
JG10-08	*ICEPca3*	NZ_CP007744.1			MOBC
JG10-08	*ICEPca1*		tRNA-Phe	Tyr	TraI
21A	*ICEPca1*	NZ_CP009125.1			

36A	*ICEPca1*	NZ_CP024956.1			
36A	*ICEPca4*		tRNA-Asn		MOBC

Pectobacterium carotovorum

Strain	Element	RefSeq	Insertion site	Integrase	Relaxase
JR1.1	*ICEPcc1*	NZ_CP034237.1	tRNA-Asn	Phage	MOBC
BP201601.1	*ICEPcc2*	NZ_CP034236.1		Tyr	

Agrobacterium tumefaciens

Strain	Element	RefSeq	Insertion site	Integrase	Relaxase
1D1609 (Cromossomo I)	*ICEAtu1*	NZ_CP026924.1	guaA	Tyr	MOBP
12D1 (Cromossomo circular)	*ICEAtu2*	NZ_CP033031.1	tRNA-Met		-
12D1 (Cromossomo linear)	*ICEAtu3*	NZ_CP033032.1		Recombinase	MOBP
186(Cromossomo circular)	*ICEAtu4*	NZ_CP042274.1	tRNA-Arg		

Pseudomonas syringae

Strain	Element	RefSeq	Insertion site	Integrase	Relaxase
B728a	*ICEPsy1*	NC_007005.1			
Shaanxi_M228	*ICEPsy2*		tRNA-Pro		
Shaanxi_M228	*ICEPsy3*	NZ_CP032631.1			
Shaanxi_M228	*ICEPsy4*		tRNA-Lys		
Shaanxi_M228	*ICEPsy5*				
NZ-45 (ICMP 20586)	*ICEPsy2*				MOBH
NZ-45 (ICMP 20586)	*ICEPsy6*				
NZ-45 (ICMP 20586)	*ICEPsy7*	NZ_CP017007.1		Tyr	
NZ-45 (ICMP 20586)	*ICEPsy8*				
MAFF212063	*ICEPsy9*	NZ_CP024712.1	tRNA-Pro		
MAFF212063	*ICEPsy1*0				-
ICMP 18708	*ICEPsy2*	NZ_CP012179.1			
ICMP 18708	*ICEPsy7*				MOBH
ICMP 18884	*ICEPsy2*	NZ_CP011972.2			

ICMP 18884	*ICEPsy7*				
CRAFRU14.08	*ICEPsy2*				
CRAFRU14.08	*ICEPsy1*1	NZ_CP019732.1	tRNA-Lys		
CRAFRU14.08	*ICEPsy1*2				
NZ-47	*ICEPsy2*				
NZ-47	*ICEPsy7*	NZ_CP017009.1	tRNA-Pro		
NZ-47	*ICEPsy1*3				
CRAFRU12.29	*ICEPsy2*				
CRAFRU12.29	*ICEPsy1*1	NZ_CP019730.1	tRNA-Lys		
CRAFRU12.29	*ICEPsy7*		tRNA-Pro		
P155/P220	*ICEPsy1*4	NZ_CP032871.1	tRNA-Lys		
P155/P220	*ICEPsy2*				
CFBP6109	*ICEPsy1*5	NZ_LT963391.1			
CFBP3840	*ICEPsy1*6	NZ_LT963409.1	tRNA-Pro		
HS191	*ICEPsy1*7	NZ_CP006256.1			
PP1	*ICEPsy1*8	NZ_CP034078.1			
CC1557	*ICEPsy1*9	NZ_CP007014.1	tRNA-Gly	Phage	
UB303	*ICEPsy2*0	NZ_CP047267.1	tRNA-Ile	Tyr	-

Xanthomonas campestris

**Strain**	**Element**	**RefSeq**	**Insertion site**	**Integrase**	**Relaxase**
B100	*ICEXca1*	NC_010688.1	tRNA-Arg		
3811	*ICEXca1*	NZ_CP025750.1		Phage	MOBH
CN03	*ICEXca2*	NZ_CP017308.1	tRNA-Gly		

Xylella fastidiosa

**Strain**	**Element**	**RefSeq**	**Insertion site**	**Integrase**	**Relaxase**
9a5c	*ICEXfa1*	NC_002488.3	ABC-F family ATP-binding cassette	Tyr	
Hib4	*ICEXfa2*	NZ_CP009885.1	tRNA-Gly	Tyr	MOBH

J1a12	*ICEXfa3*	NZ_CP009823.1	tRNA-Lys	Tyr	
U24D	*ICEXfa1*	NZ_CP009790.1	ABC-F family ATP-binding cassette	Tyr	
Ralstonia pseudosolanacearum					
**Strain**	**Element**	**RefSeq**	**Insertion site**	**Integrase**	**Relaxase**
GMI1000	*Tn4371*	NC_003295.1			
RS476	*Tn4371*	NZ_CP021762.1	purM	Ser	
CRMRs218	*Tn4371*	NZ_CP021764.1			
FJAT-91	*ICERps1*	NZ_CP016612.1			
FQY_4	*ICERps2*	NC_020799.1	tRNA-Lys		MOBP
YC40-M	*ICERps2*	NZ_CP015850.1			
HA4I	*ICERps3*	NZ_CP022481.1	tRNA adenosine	Tyr	
UW386	*ICERps4*	NZ_CP039339.1			
FJAT91-F1	*ICERps1*	NZ_CP056083.1	tRNA-Lys		
FJAT91-F8	*ICERps1*	NZ_CP056085.1			
Ralstonia syzyzii					
**Strain**	**Element**	**RefSeq**	**Insertion site**	**Integrase**	**Relaxase**
T98	*ICERsy1*	NZ_CP022759.1	guaA	Tyr	MOBP
SL3175	*ICERsy1*	NZ_CP022788.1		Tyr	

Supplementary table 15: Putative significant cargo genes carried by ICEs characterized in this work

Element	Gene	Putative Function
ICEAtu1	Alkene reductase<br>Glutathione S-transferase	Adaptation
	Cysteine hydrolase<br>Glycosidase	Virulence
ICEAtu3	Universal stress protein<br>aqpZ	Adaptation
	Diguanylate cyclase<br>Invasion associated locus B	Virulence
	L.D - transpeptidase	Antibiotic Resistance
ICEAtu4	Endo-1,4-beta-xylanase<br>Isochorismatase family protein	Virulence
	MBL fold metallo-hydrolase	Antibiotic Resistance
ICEDda1	tssI - VgrG<br>Hcp effector	Virulence
	TerB	Tellurium resistance
	EexN	Entry exclusion
ICEDda2	PAAR domain containing protein<br>tssI - VgrG<br>Hcp effector	Virulence
ICEDso1	tssI - VgrG<br>Hcp effector<br>Amidohydrolase	Virulence
ICEPca1	Coronafacic acid dehydratase<br>Coronafacic acid synthetase<br>Coronafacic acid synthetase component<br>Coronafacate ligase	Virulence
	Type I polyketide synthase	Secondary metabolism
ICEPca2	EexN	Entry exclusion
	Phospholipase D	Virulence
ICEPca3	Glutathione S-transferase	Adaptation
	MBL fold metallo-hydrolase	Antibiotic Resistance
ICEPca4	Arginase family protein	Virulence
	MipA/OmpV family protein	Antibiotic Resistance
	EexN	Entry exclusion
ICEPcc1	EexN	Entry exclusion
ICEPcc2	Glutathione peroxidase	Antioxidant Resistance
	MBL fold metallo-hydrolase	Antibiotic Resistance
ICEPsy1	Copper resistance cluster	Metal resistance

	Arsenic resistance cluster	Arsenic resistance
	CrpP family protein	Antibiotic Resistance
ICEPsy2	Tellurium resistance cluster	Tellurium resistance
	Trehalose phosphatase	Adaptation
	PSPTO0907<br>Type III secretion system effector phosphothreonine lyase<br>Cellulase family glycosylhydrolase<br>NUDIX hydrolase<br>Alginate lyase<br>Glycoside hydrolase family 32 protein<br>Type III effector HopR1<br>Effector protein<br>Type III effector<br>Type III chaperone protein ShcF	Virulence
ICEPsy3	DsbA family protein<br>Type VI secretion system tube protein Hcp<br>SEC-C domain-containing protein	Virulence
	CrpP family protein	Antibiotic Resistance
ICEPsy4	Arsenic resistance cluster	Arsenic resistance
	Ultraviolet light resistance protein RulA<br>umuC	UV Resistance
	CrpP family protein	Antibiotic Resistance
ICEPsy5	AvrRpm1	Avirulence
	Type III chaperone protein ShcF<br>Type III effector<br>Peptidase C55<br>Type III secretion system effector	Virulence
ICEPsy6	PapB Adhesin biosynthesis transcription regulatory protein	Virulence
	CrpP family protein	Antibiotic Resistance
ICEPsy7	TonB-dependent siderophore receptor	Adaptation
	AvrRpm1	Avirulence
	Peptidase C55<br>Type III secretion system effector	Virulence
ICEPsy8	Copper resistance cluster	Metal resistance
	Arsenic resistance cluster	Arsenic resistance
	CrpP family protein	Antibiotic Resistance
ICEPsy9	Type III effector	Virulence
	HopAU1	Avirulence
	CrpP family protein	Antibiotic Resistance
ICEPsy10	gstA<br>MOSC domain-containing protein	Adaptation
	PSPTO0907<br>Type III secretion system effector phosphothreonine lyase<br>Cellulase family glycosylhydrolase<br>Type VI secretion system effector	Virulence

	Type III effector Cluster<br>Type III chaperone protein ShcF<br>CesT family type III secretion system chaperone<br>YopT-type cysteine protease domain-containing protein<br>C58 family peptidase<br>Syringolide biosynthetic protein AvrD1<br>Type III effector HopAM1<br>Type III effector HopF2	
	AvrPphF	Avirulence
ICEPsy11	Arsenic resistance cluster	Arsenic resistance
	CrpP family protein	Antibiotic Resistance
	Ultraviolet light resistance protein RulA	UV Resistance
	TcpQ domain-containing protein	Virulence
ICEPsy12	TonB-dependent siderophore receptor	Adaptation
	Type III secretion system effector protein<br>Peptidase C55	Virulence
ICEPsy13	Copper resistance cluster	Metal resistance
	CrpP family protein	Antibiotic Resistance
ICEPsy14	Copper resistance cluster	Metal resistance
	Arsenic resistance cluster<br>CrpP family protein	Arsenic resistance<br>Antibiotic Resistance
	PhzF family phenazine biosynthesis isomerase	Virulence
ICEPsy16	CrpP family protein	Antibiotic Resistance
	Glycoside hydrolase family 68 protein<br>Glycoside hydrolase family 10 protein<br>Lytic murein transglycosylase	Virulence
ICEPsy17	CrpP family protein	Antibiotic Resistance
	salA	Virulence
ICEPsy18	CrpP family protein<br>MupB	Antibiotic Resistance
	Alcohol dehydrogenase catalytic domain-containing protein<br>Lytic murein transglycosylase<br>CesT family type III secretion system chaperone<br>AvrPphF family type III effector	Virulence
ICEPsy19	MBL fold metallo-hydrolase<br>Lysozyme inhibitor LprI	Antibiotic Resistance
**Tn4371**	Glutathione S-transferase	Adaptation
	Cysteine hydrolase family protein	Virulence
**ICERps.1**	Gamma-glutamylcyclotransferase	Adaptation
**ICERps.3**	Glutathione S-transferase	Adaptation
**ICERsy.1**	Amidohydrolase	Virulence
	Superoxide dismutase	Antioxidant Resistance
ICEXca2	AvrXccC effector<br>AvrXacE1 effector<br>pPg6L3/ AvrA	Avirulence

	Lytic murein transglycosylase	Virulence
	TauD/TfdA family dioxygenase<br>sulfotransferase<br>Coproporphyrinogen III oxidase<br>Inositol hexakisphosphate	Adaptation
ICEXfa1	lpxO	Resistance
	Virulence factor	Virulence
ICEXfa2	MBL fold metallo-hydrolase	Antibiotic Resistance
	Superoxide dismutase [Fe]	Antioxidant Resistance
	Efflux transporter outer membrane subunit<br>Efflux RND transporter permease subunit	Antibiotic Resistance
	Phosphoadenosine phosphosulfate reductase familyprotein<br>Coproporphyrinogen III oxidase	Adaptation
ICEXfa3	lpxO	Resistance
	UDP-3-O-(3-hydroxymyristoyl) glucosamine N-acyltransferase	Antibiotic Resistance
	Virulence factor	Virulence
	EexN	Entry exclusion

Supplementary table 16: Elements of this work described in the literature

Element	Name	Reference
ICEDda1	Tn4371	Toussaint et al., 2003
ICEPca1	HAI2	Bell et al., 2004
ICEXfa2	-	Denancé et al., 2019
ICEPsy1	PsyrGI-6	Feil et al, 2005
ICEPsy6	Pac_ICE1	Butler et al, 2013
ICEPsy4	Pac_ICE2	Butler et al, 2013
ICEPsy8	Psa_{NZ45}ICE_Cu	Colombi et al, 2017
ICEPsy7	Psa_{NZ13}ICE_eno	Colombi et al, 2017
*ICEPsy1*3	Psa_{NZ47}ICE_Cu	Colombi et al, 2017
Tn4371	Tn4371	Salanoubat et al., 2012
ICERps1	ICERps1	Gonçalves et al., 2020
ICERps2	ICERps2	Gonçalves et al., 2020
ICERps3	ICERps3	Gonçalves et al., 2020
ICERsy1	ICERsy1	Gonçalves et al., 2020
