# Supplementary material for: Genomic analysis reveals the role of integrative and conjugative elements in plant pathogenic bacteria
Source: Mob DNA. 2022 Aug 12;13:19. doi: 10.1186/s13100-022-00275-1 (PMC9373382; doi:10.1186/s13100-022-00275-1)
Supplement: Supplementary file 2 — Additional file 2: Supplementary Figure 1. Gene clusters comparision of Agrobacterium tumefaciens ICEs. Supplementary Figure 2. Gene clusters comparision of Dickeya ICEs. Supplementary Figure 3. Gene clusters comparision of Pectobacterium carotovorum (and atrosepticum) ICEs. Supplementary Figure 4. Gene clusters comparision of group 1 Pseudomonas syringae ICEs. Supplementary Figure 5. Gene clusters comparision of group 2 Pseudomonas syringae ICEs. Supplementary Figure 6. Gene clusters comparision of group 3 Pseudomonas syringae ICEs. Supplementary Figure 7. Gene clusters comparision of RSSC ICEs. Supplementary Figure 9. Gene clusters comparision of Xylella fastidiosa ICEs. Supplementary Figure 10. ICEs identity matrix heatmaps. [file 13100_2022_275_MOESM2_ESM.pdf]

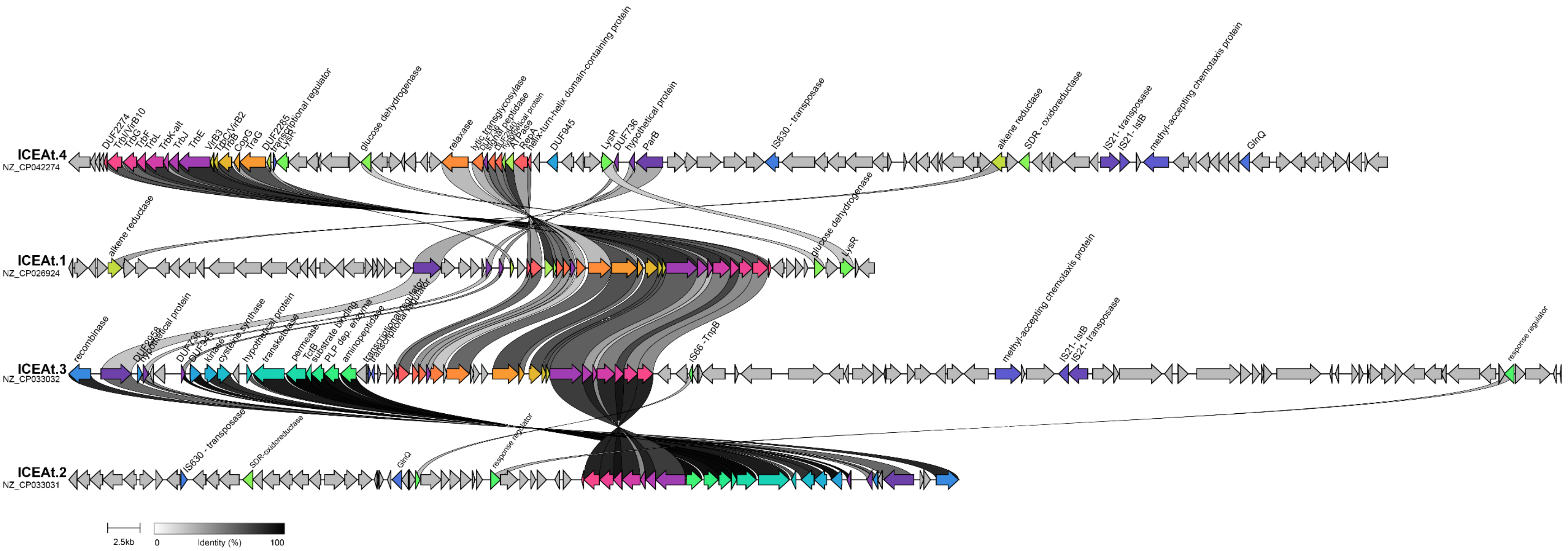

Supplementary Figure 1*:* **Gene clusters comparision of *Agrobacterium tumefaciens* ICEs.** ORFs are represented by arrows, colloured arrows represents different synthenic gene clusters, genes products are indicated in the representative ORF arrow.

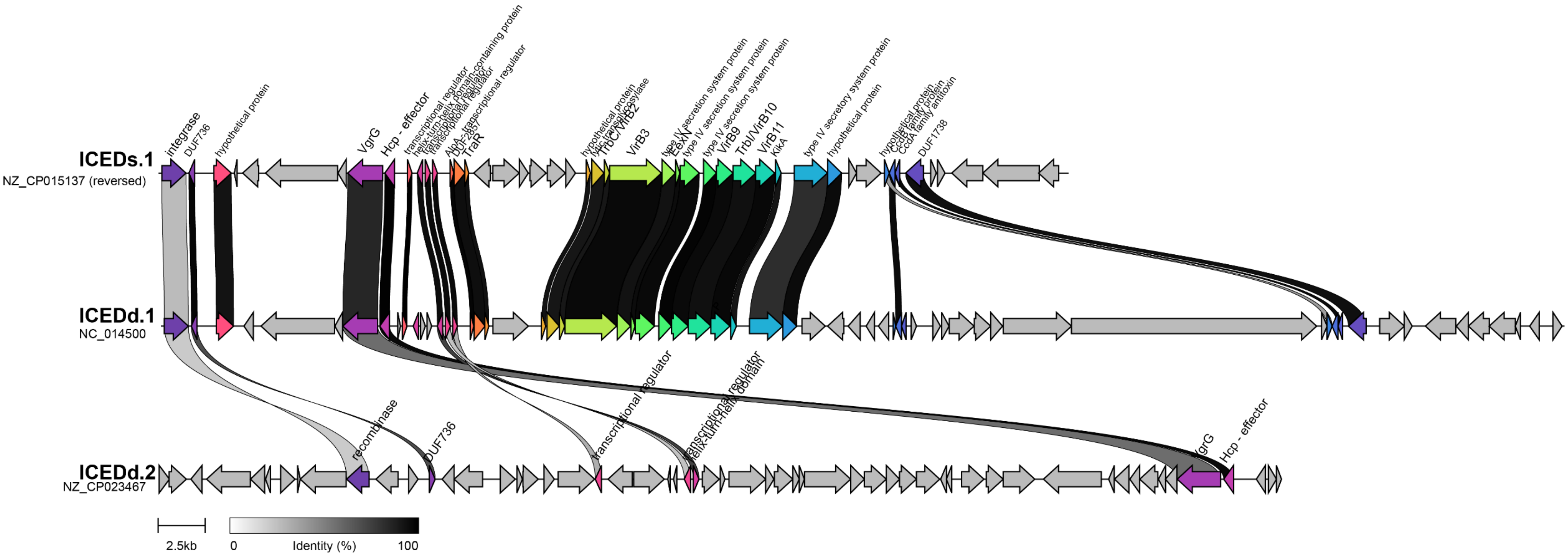

Supplementary Figure 2*: *Gene clusters comparision of *Dickeya* ICEs. ORFs are represented by arrows, colloured arrows represents different synthenic gene clusters, genes products are indicated in the representative ORF arrow.

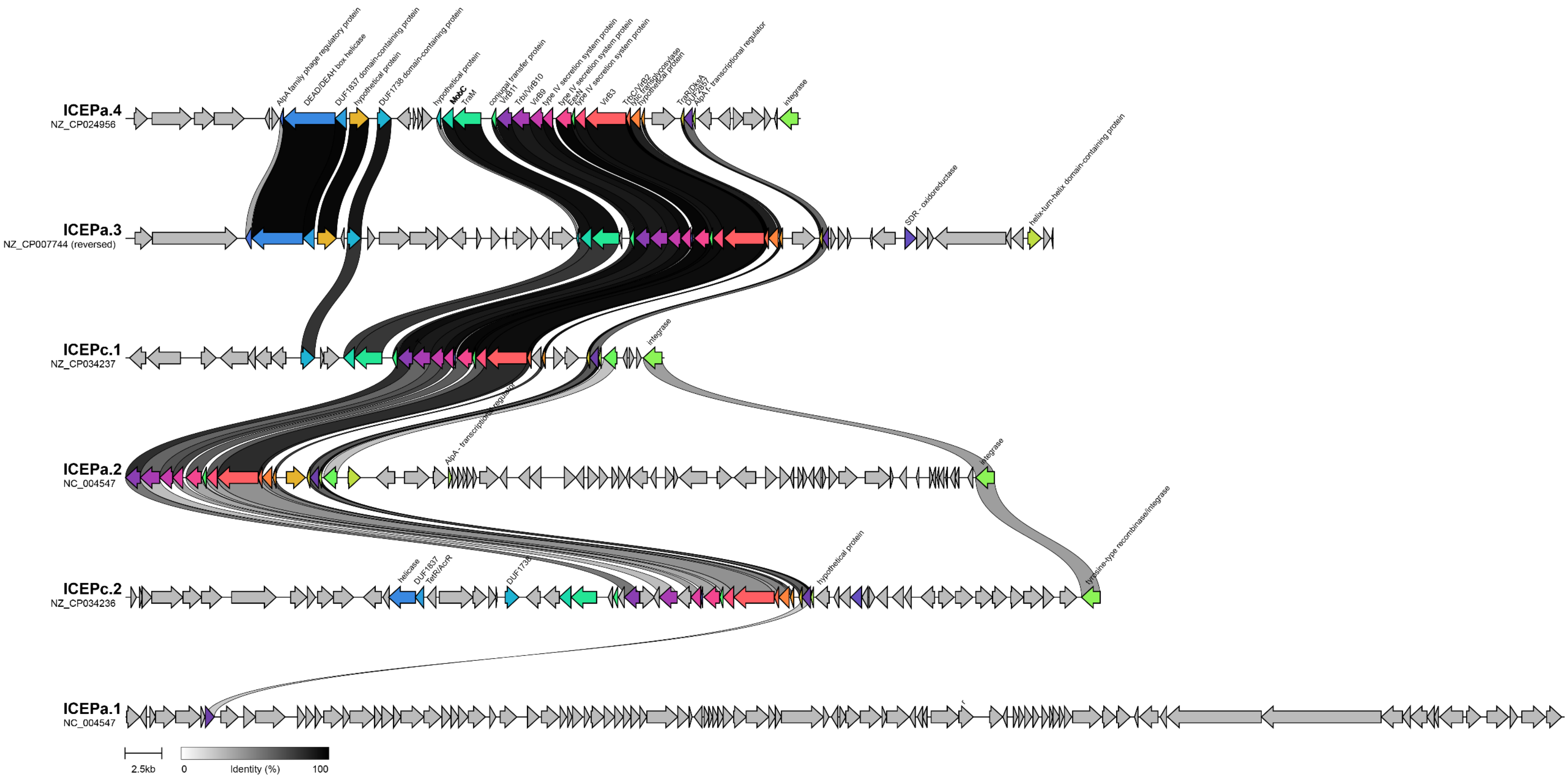

Supplementary Figure 3*:* **Gene clusters comparision of *Pectobacterium carotovorum* (and *atrosepticum*) ICEs.** ORFs are represented by arrows, colloured arrows represents different synthenic gene clusters, genes products are indicated in the representative ORF arrow.

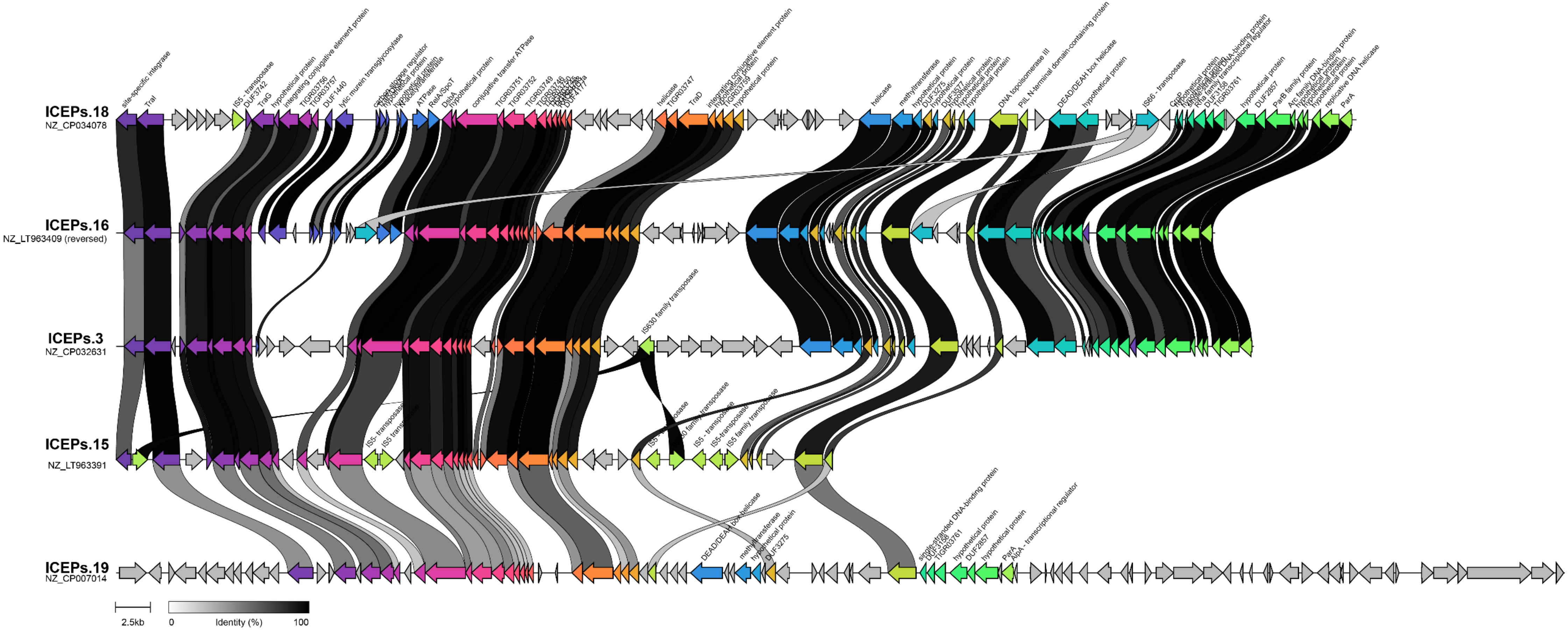

Supplementary Figure 4*:* Gene clusters comparision of group 1 *Pseudomonas syringae* ICEs. ORFs are represented by arrows, coloured arrows represents different synthenic gene clusters, genes products are indicated in the representative ORF arrow

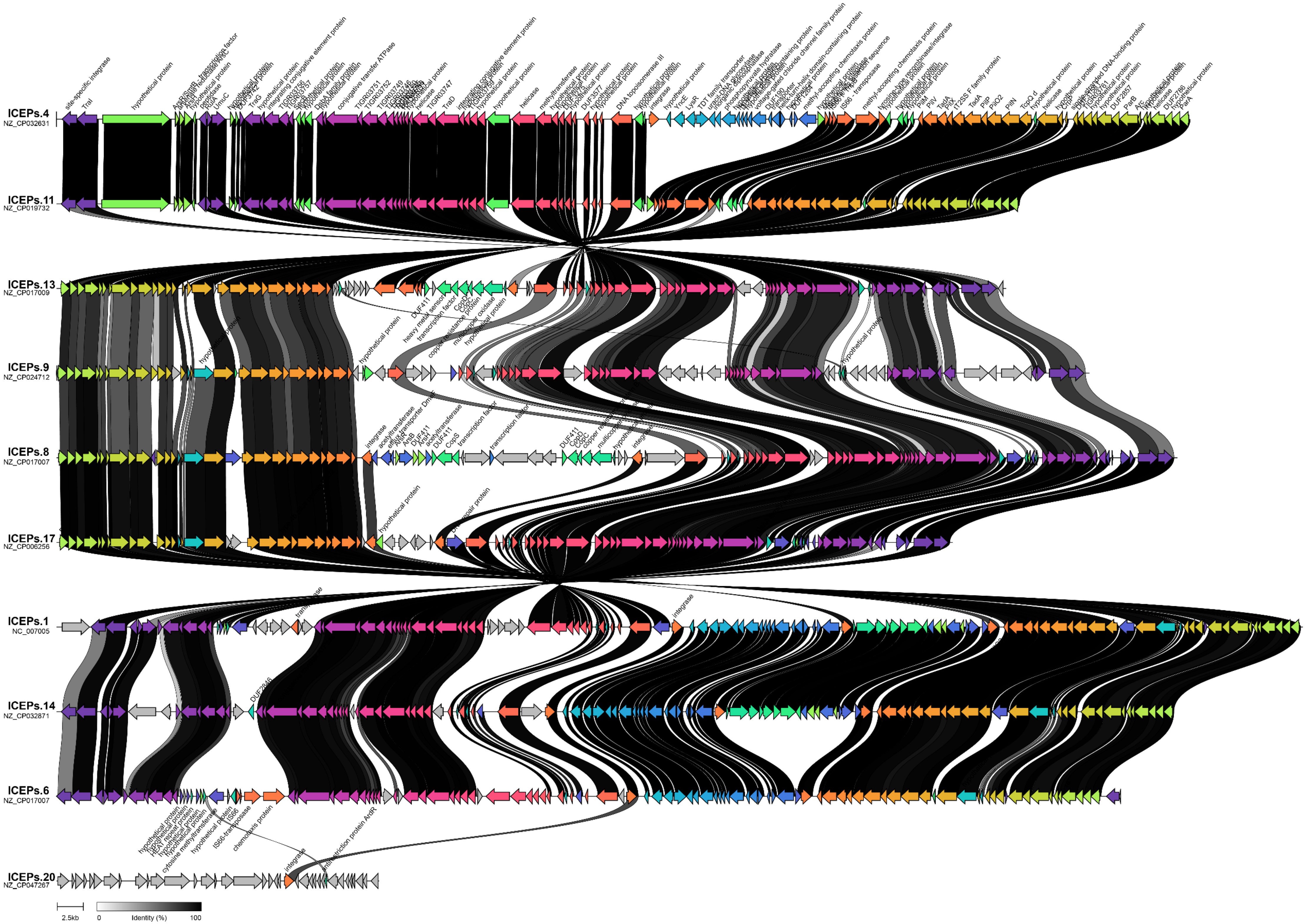
ICEPs.4
NZ_CP032631
ICEPs.11
NZ_CP019732
ICEPs.13
NZ_CP017009
ICEPs.9
NZ_CP024712
ICEPs.8
NZ_CP017007
ICEPs.17
NZ_CP006256
ICEPs.1
NC_007005
ICEPs.14
NZ_CP032871
ICEPs.6
NZ_CP017007
ICEPs.20
NZ_CP047267
2.5kb
0
Identity (%)
100

Supplementary Figure 5: Gene clusters comparision of group 2 *Pseudomonas syringae* ICEs. ORFs are represented by arrows, colloured arrows represents different synthenic gene clusters, genes products are indicated in the representative ORF arrow.

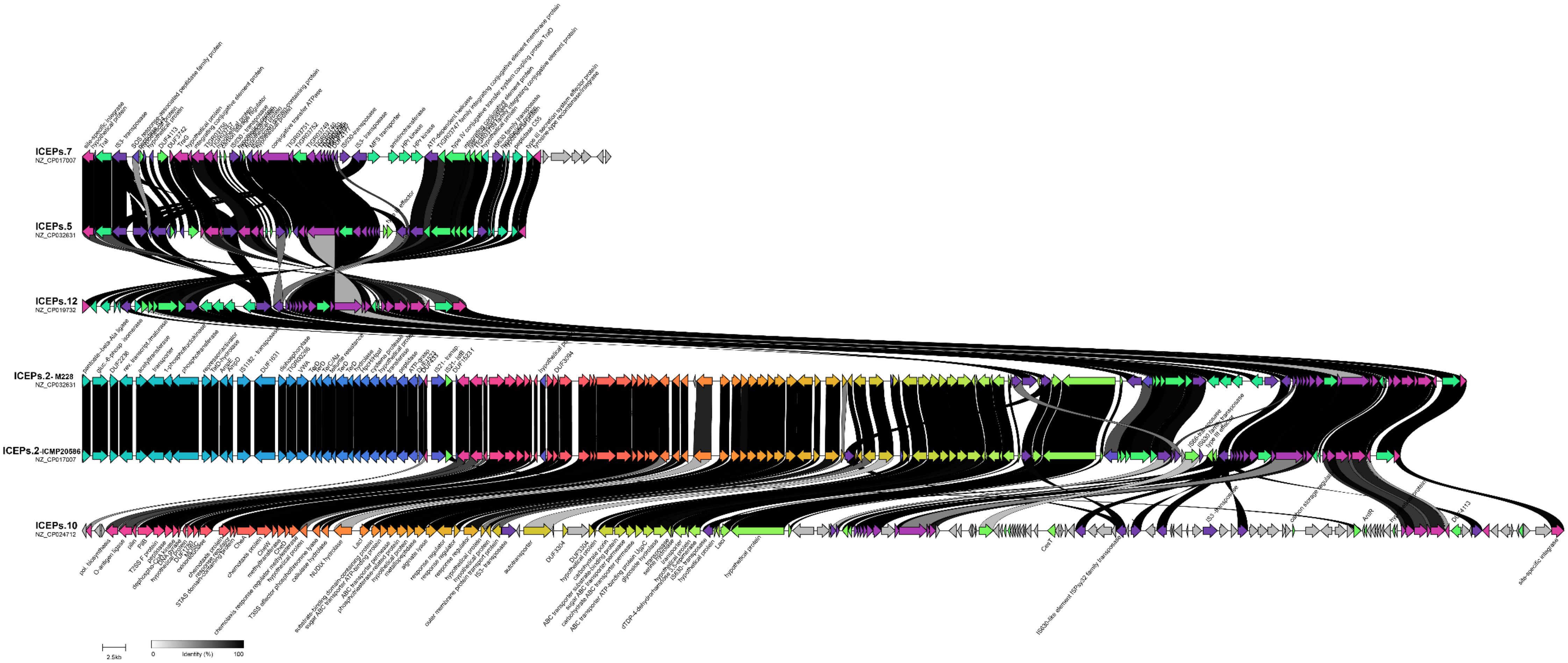

Supplementary Figure 6: Gene clusters comparision of group 3 *Pseudomonas syringae* ICEs. ORFs are represented by arrows, colloured arrows represents different synthenic gene clusters, genes products are indicated in the representative ORF arrow.

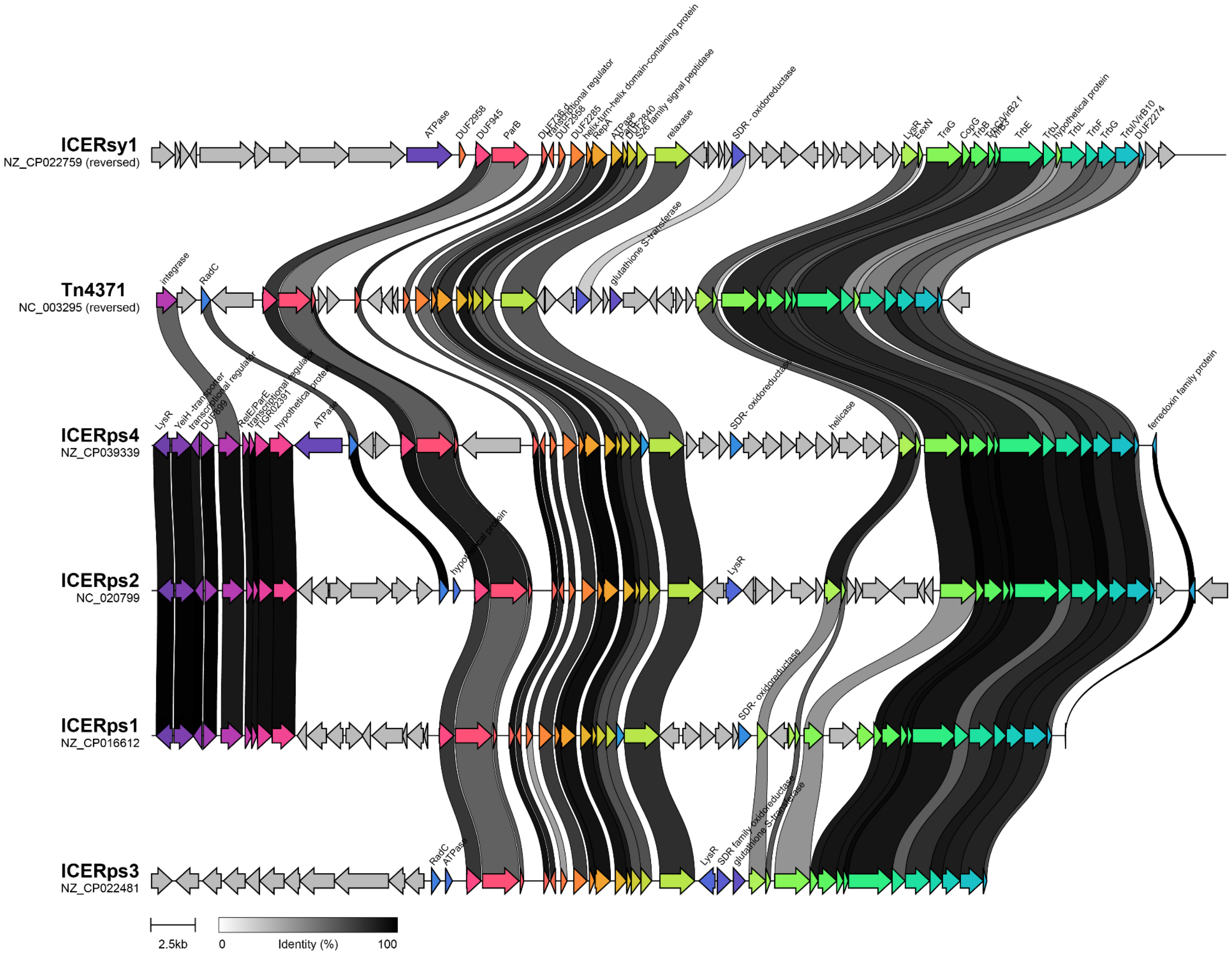

Supplementary Figure 7: Gene clusters comparision of RSSC ICEs. ORFs are represented by arrows, colloured arrows represents different synthenic gene clusters, genes products are indicated in the representative ORF arrow.

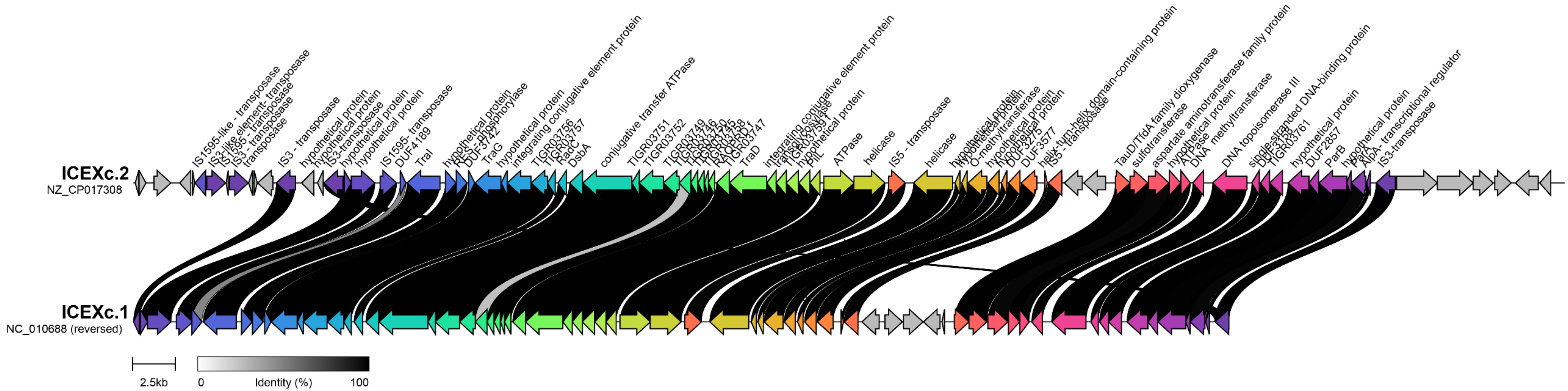

Supplementary Figure 8*:* **Gene clusters comparision of *Xanthomonas campestris* ICEs.** ORFs are represented by arrows, colloured arrows represents different synthenic gene clusters, genes products are indicated in the representative ORF arrow.rs, genes products are indicated in the representative ORF arrow.

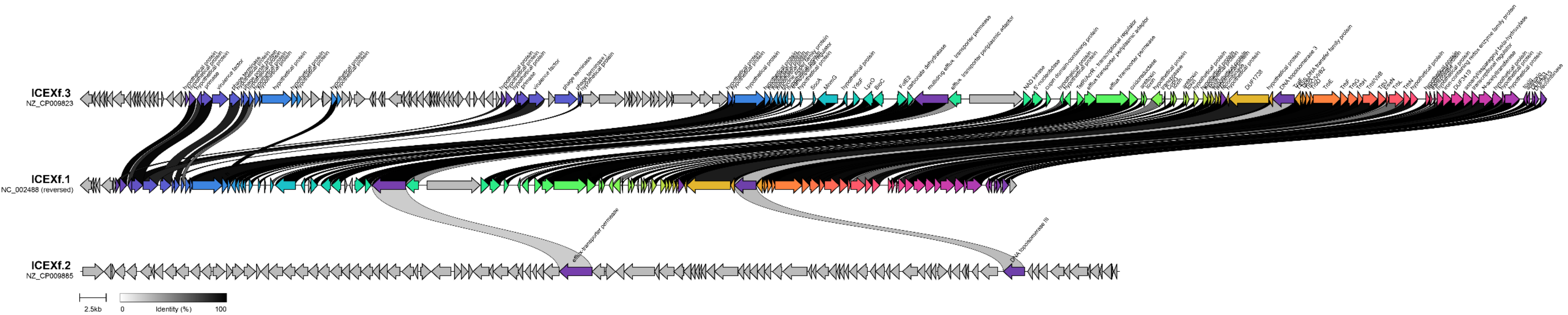

Supplementary Figure 9*:* **Gene clusters comparision of *Xylella fastidiosa* ICEs.** ORFs are represented by arrows, colloured arrows represents different synthenic gene clusters, genes products are indicated in the representative ORF arrow.rs, genes products are indicated in the representative ORF arrow.

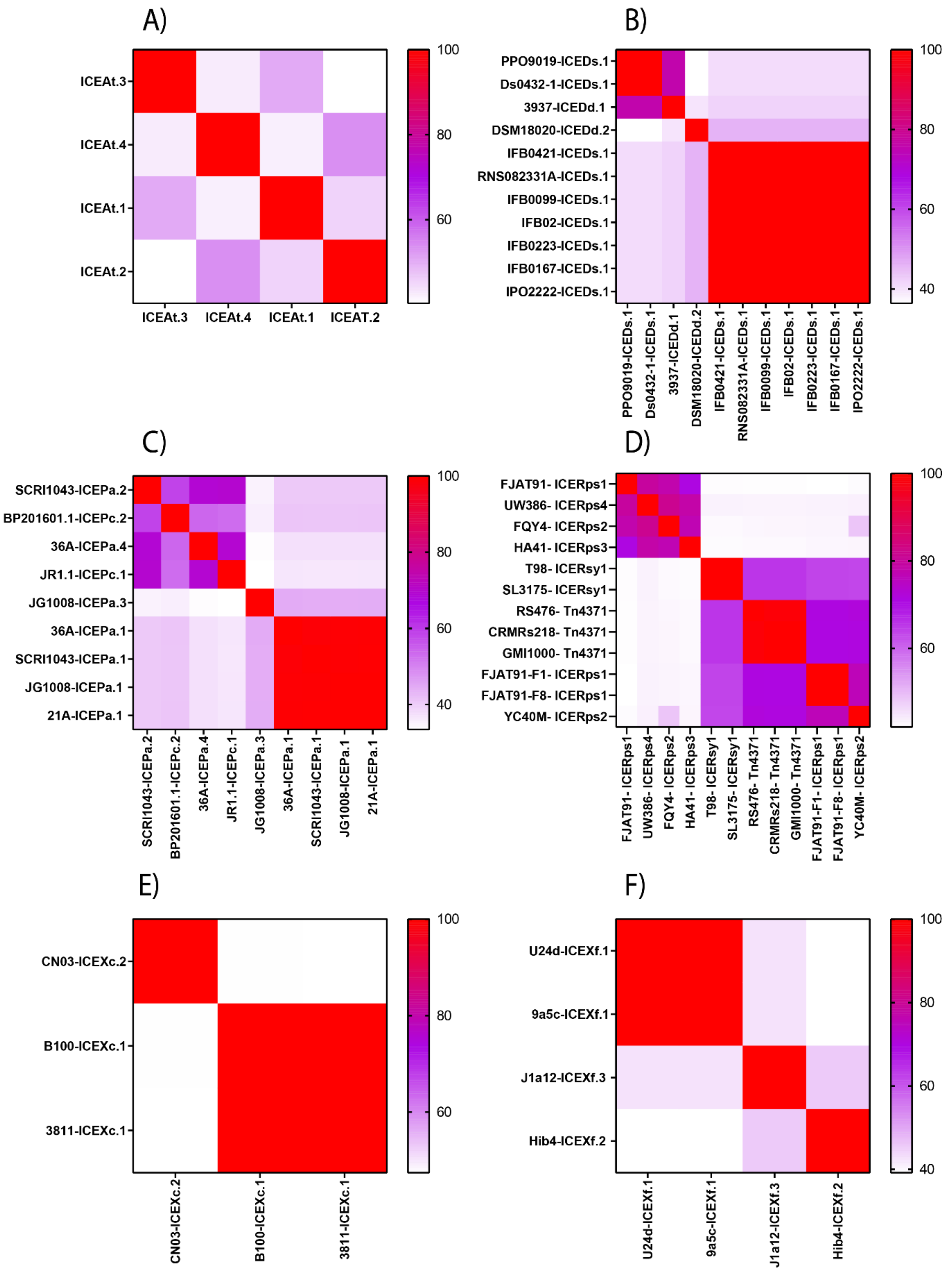

Supplementary Figure 10: ICEs identity matrix heatmaps. Heatmaps are divided by groups of bacteria; red color represents 100% of nucleotide identity A) *A. tumefaciens*; B) *Dickeya* (*dadantii* and *solani*); C) *P. carotovorum* (and *atrosepticum*); D) *R. solanacearum* species complex; E) *X. campestris*; F) *X. fastidiosa*.
